# Pulsed electromagnetic fields inhibit atherosclerosis by regulating pyroptosis through membrane tension-mediated mechanosensitive channels

**DOI:** 10.1038/s41392-025-02479-2

**Published:** 2025-11-28

**Authors:** Hongxin Cheng, Qing Zhang, Wen Zhong, Hanbin Li, Lu Wang, Shiqi Wang, Chengqi He, Chenying Fu, Quan Wei

**Affiliations:** 1 Department of Rehabilitation Medicine Center and Institute of Rehabilitation Medicine, West China Hospital, Sichuan University, Chengdu, Sichuan China; 2 Key Laboratory of Rehabilitation Medicine in Sichuan Province, Chengdu, Sichuan China; 3 State Key Laboratory of Biotherapy and National Clinical Research Center for Geriatrics, West China Hospital, Sichuan University, Chengdu, Sichuan China; 4 Geriatric Health Care and Medical Research Center, National Clinical Research Center for Geriatrics, West China Hospital, Sichuan University, Chengdu, Sichuan China

**Keywords:** Medical research, Cardiology

## Abstract

Atherosclerosis serves as the core pathological basis of cardiovascular, cerebrovascular, and peripheral arterial diseases, posing a serious threat to human health. However, current mainstream treatments such as statin drugs and stent implantation are associated with significant side effects or limited efficacy, highlighting the urgent need for new therapeutic strategies. Pulsed electromagnetic fields (PEMFs), due to their noninvasive nature and anti-inflammatory properties, show potential in the treatment of atherosclerosis. This study utilized ApoE-/- mice, ApoE-/-NLRP3-/- knockout mice, human umbilical vein endothelial cells (HUVECs), human aortic endothelial cells (HAECs), and human plasma samples for experiments, revealing significant endothelial cell (EC) inflammation and pyroptosis during the progression of atherosclerosis. PEMFs were found to effectively inhibit the activation of the NLRP3 inflammasome, reduce plaque formation, and delay the progression of atherosclerosis. Proteomic analysis of plasma from atherosclerosis patients further indicated elevated expression levels of proteins related to inflammation and pyroptosis, with particularly notable changes in membrane proteins. Mechanistic studies demonstrated that PEMFs improve mitochondrial dysfunction in ECs by regulating membrane tension and the mechanosensitive tension-mediated transient receptor potential vanilloid 4 (TRPV4) channels, thereby reducing pyroptosis. This discovery not only reveals a novel mechanobiological pathway but also provides a solid theoretical foundation for the development of PEMF-based therapies for atherosclerosis.

**Figure:**
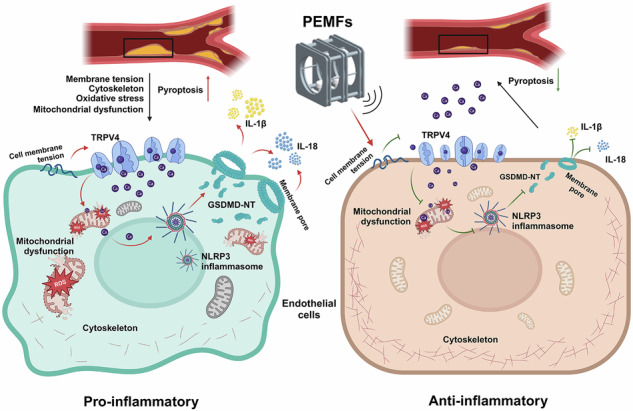
Schematic diagram of the mechanism by which PEMFs treat atherosclerosis (created in BioRender). Wei, B. (2025) https://BioRender.com/undefined).

Schematic diagram of the mechanism by which PEMFs treat atherosclerosis (created in BioRender). Wei, B. (2025) https://BioRender.com/undefined).

## Introduction

Atherosclerosis is a chronic inflammatory disease of complex pathogenesis, characterized by the involvement of multiple cell types and factors.^1^ According to statistics from the World Health Organization, atherosclerotic vascular diseases account for one-third of deaths worldwide.^2^ The formation of atherosclerotic plaques results from interactions among vascular ECs, monocytes/macrophages, and smooth muscle cells and the activation of numerous inflammatory factors.^3,4^ Among these factors, as a pivotal initiator and central driver of atherosclerosis, EC inflammation can induce lipid deposition and the formation of cholesterol crystals,^5^ thereby exacerbating the progression of atherosclerosis.^4^ The conceptualization of atherosclerosis as a chronic inflammatory disease has evolved through cumulative research efforts. Ross first articulated this framework in 1999,^6^ with subsequent work by Hansson^7^ and Libby^8^ further refining and validating this model. Since then, a growing number of studies have confirmed that inflammation is a hallmark throughout the entire course of atherosclerosis. Therefore, with increasing research, anti-inflammatory therapy has become a new way to explore the treatment of atherosclerosis.

In recent years, many inflammation-related studies have focused on pyroptosis, an inflammation-related form of programmed cell death.^9^ The NOD-like receptor thermal protein domain associated protein 3 (NLRP3) inflammasome is a classic upstream regulator of pyroptosis.^10^ As a multiprotein complex, the NLRP3 inflammasome consists of three core components: NLRP3 (the sensor protein that recognizes intracellular danger signals), apoptosis-associated speck-like protein containing a C-terminal caspase recruitment domain (CARD) (ASC, the adaptor protein that mediates complex assembly), and pro-cysteinyl aspartate specific proteinase-1 (caspase-1) (the effector zymogen, containing an N-terminal CARD).^11^ The NLRP3 inflammasome is typically activated by pathogen-associated molecular patterns (PAMPs, such as microbial toxins, viral RNA, and surface components of bacteria) and damage-associated molecular patterns (DAMPs, including ATP, uric acid crystals, beta-amyloid peptides, and aluminum adjuvants).^10,12^ Upon activation, NLRP3 recruits ASC via pyrin domain interactions, and ASC further aggregates to form speckles that recruit procaspase-1 through CARD–CARD domain binding. This assembly process triggers self-cleavage of procaspase-1 into active caspase-1, which in turn cleaves proinflammatory cytokine precursors (pro-interleukin (IL)-1β, pro-IL-18) into their mature and secreted forms.^12^ Concurrently, active caspase-1 cleaves gasdermin D (GSDMD) into N-terminal fragments (GSDMD-NT), which oligomerize to form pores in the cell membrane, inducing pyroptosis and facilitating the release of mature IL-1β and IL-18 to amplify the inflammatory response.^10^ Recent studies indicate a strong association between pyroptosis and atherosclerosis. In the early stage of atherosclerosis, EC dysfunction and death are key drivers of disease initiation. EC pyroptosis, in particular, can induce cell dysfunction, cell death, and the release of inflammatory cytokines, thereby accelerating atherosclerotic plaque formation.^13^

As a noninvasive physical therapy, PEMFs play an important role in treating chronic diseases. Many studies have reported that PEMFs can inhibit the acute and chronic inflammatory response in rheumatoid arthritis,^14^ osteoarthritis,^15^ and other diseases.^16,17^ In addition, PEMF intervention can promote the recovery of vascular functional homeostasis.^14^ Javadzadegan et al. confirmed through computational fluid dynamics simulations of patient coronary artery models that magnetic fields could improve hemodynamic disorders and reduce blood viscosity, with this effect being particularly evident in cases of moderate to severe stenosis.^18^ Interestingly, Ramasamy Selvam et al. demonstrated that PEMFs may regulate inflammation by altering the intracellular calcium concentration in their experimental setting.^19^ The regulatory effect of PEMFs on vascular function has attracted clinical attention. A number of clinical studies on hypertensive patients have provided important evidence for its potential application value; for example, a randomized controlled trial involving hypertensive patients revealed that PEMF intervention could significantly reduce patients’ systolic and diastolic blood pressure while improving flow-mediated dilation, suggesting that it may regulate blood pressure by improving endothelium-dependent vasodilation.^20^ Another study on mild to moderate metabolic syndrome further confirmed that long-term PEMF intervention could reduce resting and postexercise blood pressure, accompanied by an increase in serum nitric oxide (NO) levels, indicating that it may exert vascular protective effects by enhancing NO utilization.^21^ This clinical evidence indicates that PEMFs can participate in blood pressure regulation by improving endothelial function and regulating vascular tone, providing an important reference for understanding their role in vascular diseases such as atherosclerosis. However, the therapeutic use of PEMFs for atherosclerosis is still in its infancy. Therefore, in this study, we focused on the impact of PEMFs on EC membrane tension, inflammation, and mitochondrial function in atherosclerosis, aiming to reveal the potential mechanism of their vascular protection at the cellular and molecular levels and provide more comprehensive theoretical support for the clinical transformation and application of PEMFs.

Our results revealed that PEMFs mitigate plaque formation and delay the progression of atherosclerosis by suppressing NLRP3 inflammasome activation. Importantly, we further elucidated that this inhibition is achieved by modulating cell membrane tension and the TRPV4 mechanosensitive channel to ameliorate mitochondrial dysfunction. Collectively, our findings provide novel mechanistic insights into the therapeutic role of PEMFs in atherosclerosis, highlighting that PEMFs can inhibit the progression of atherosclerosis by improving membrane tension and ultimately downregulating NLRP3 inflammasome expression.

## Results

### PEMFs (15 Hz, 1.5 mT) exhibit superior efficacy in suppressing atherosclerotic plaque formation

Building on previous evidence of the beneficial effects of PEMFs on cardiovascular diseases and the limited research on their application in atherosclerosis, we systematically evaluated three PEMF parameter sets in atherosclerotic mouse models to identify optimal treatment conditions: (1) 5 Hz/0.5 mT/60 min/3 weeks (PEMFs I group), (2) 15 Hz/1.5 mT/60 min/3 weeks (PEMFs II group), and (3) 30 Hz/3.0 mT/60 min/3 weeks (PEMFs III group). Oil red O staining revealed that all three PEMF regimens significantly attenuated atherosclerotic plaque formation, with the most pronounced effect in the HFD+PEMFs II group (supplementary Fig. 1a, b). The serum levels of the proinflammatory factors IL-1β and IL-18 also decreased across all PEMF groups, with the strongest suppression again observed in the HFD+PEMFs II group (supplementary Fig. 1c, d). On the basis of these results, the PEMFs II protocol (15 Hz/1.5 mT/60 min/3 weeks) was selected for subsequent in vivo experiments.

### PEMFs inhibit the formation of atherosclerotic plaques by attenuating pyroptosis and inflammation

**Fig. 1:**
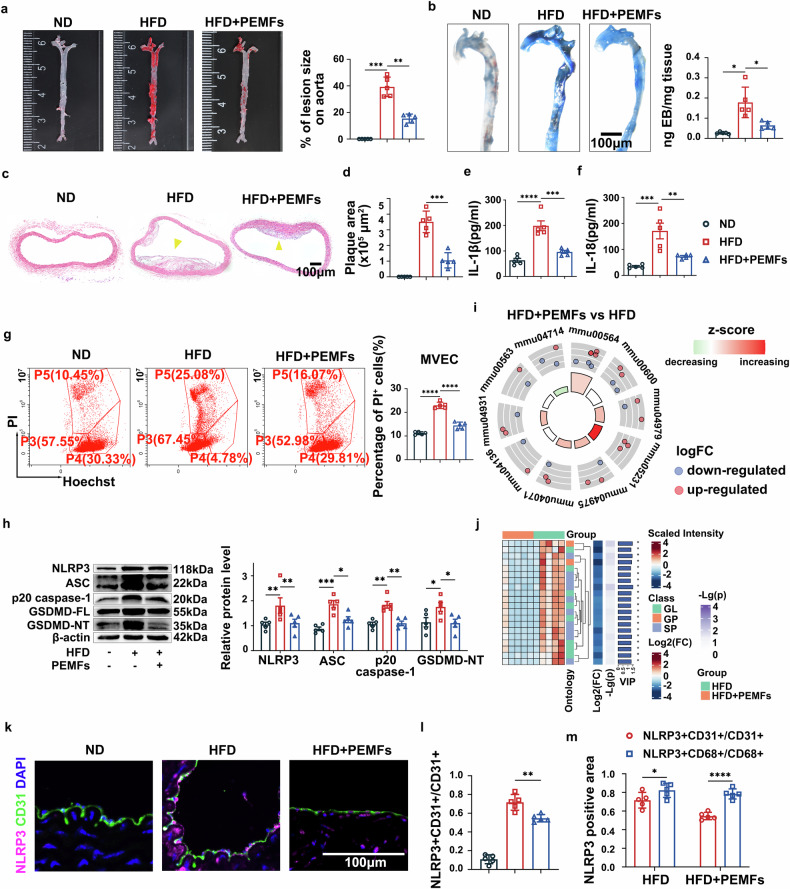
PEMFs inhibit the formation of atherosclerotic plaques by attenuating pyroptosis and inflammation. **a** Oil red O staining was used to determine the lesion burden in the aorta in the ApoE-/- mouse groups: the normal chow diet (ND) group, high-fat diet (HFD, 15 weeks) group, and HFD+PEMFs (15 Hz, 1.5 mT, 1 h/day, 3 weeks) group; *n* = 5 mice per group. **b** Evans blue staining was used to assess aortic permeability under the same experimental conditions; *n* = 5 mice per group. Scale bar = 100 μm. **c**, **d** HE staining was used to visualize the plaque area (yellow arrows) in the aortic arch, and the results were quantified; *n* = 5 mice per group. Scale bar = 100 μm. **e**, **f** Serum levels of IL-1β and IL-18 were detected by ELISA; *n* = 5 mice per group. **g** Pyroptosis was assessed by flow cytometry via Hoechst 33342/PI staining in MVECs. **h** Western blot analysis of NLRP3, ASC, p20 caspase-1, and GSDMD-NT in aortic tissues; *n* = 5 mice per group. **i** Untargeted lipidomic analysis of aortic tissues from all experimental groups. KEGG pathway analysis of the top 10 differential lipids between the HFD + PEMF and HFD groups. Dots represent metabolites (blue: downregulated; red: upregulated). The pathway descriptions are described as follows: mmu00564: Glycerophospholipid metabolism; mmu00600: Sphingolipid metabolism; mmu04979: Cholesterol metabolism; mmu05231: Choline metabolism in cancer; mmu04975: Fat digestion and absorption; mmu04071: Sphingolipid signaling pathway; mmu04136: Autophagy–other; mmu04931: Insulin resistance; mmu00563: Glycosylphosphatidylinositol (GPI)−anchor biosynthesis; mmu04714: Thermogenesis; *n* = 5 mice per group. **j** Hierarchical clustering of the top 20 significantly altered lipids between the HFD + PEMF group and the HFD group, classified by LIPID MAPS: GL (glycerolipid), SP (sphingolipid), and GP (glycerophospholipid), *n* = 5 mice per group. **k** Double immunostaining of NLRP3 and CD31 in atherosclerotic lesions among different groups of mice; *n* = 5 mice per group. Scale bar = 100 μm. **l** Manders’ coefficient was used to evaluate the NLRP3-positive area within CD31-positive regions in the aortic arch plaques; *n* = 5 mice per group. **m** Quantitative comparison of NLRP3 colocalization with CD31 (ECs) and CD68 (macrophages) in the same mice; *n* = 5 mice per group. All the data represent biological replicates. The measured data are presented as the mean ± SEM. Statistical significance was assessed by one-way ANOVA with Tukey’s multiple comparison test. *p < 0.05, **p < 0.01, ***p < 0.001, ****p < 0.0001

Compared with the HFD group (15 weeks of HFD feeding), the HFD+PEMFs group exhibited significantly slower the progression of atherosclerosis in ApoE-/- mice. This was evidenced by a reduced lesion burden (Oil red O, Fig. 1a), decreased vascular permeability (Evans blue, Fig. 1b), and diminished plaque area in the aortic arch (HE, Fig. 1c, d), indicating that PEMFs treat atherosclerosis by inhibiting plaque formation and restoring endothelial barrier function.

PEMF treatment also had potent anti-inflammatory effects, significantly reducing the serum levels of IL-1β, IL-18, IL-6, MCP-1, and TNF-α (Fig. 1e, f, supplementary Fig. 2a–c). To explore the observed potent anti-inflammatory effects of PEMFs in atherosclerotic mice, we performed western blot (WB) analysis to investigate inflammasome modulation. Quantitative protein expression analysis revealed that these effects were associated with selective downregulation of the NLRP3 inflammasome pathway: the levels of the NLRP3, ASC, and p20 caspase-1 were significantly decreased (Fig. 1h), whereas the protein levels of NLRC4 and AIM2 remained unchanged (supplementary Fig. 2d–f). Furthermore, the level of GSDMD-NT, the executor of pyroptosis, was also reduced (Fig. 1h). Flow cytometric analysis of propidium iodide (PI)-positive staining revealed that PEMFs significantly reduced pyroptosis in mouse vascular endothelial cells (MVECs) (Fig. 1g). Transmission electron microscopy (TEM) imaging revealed obvious pyroptotic morphology, including cell swelling, balloon-like bubble structures, cell membrane pores and discontinuities, in the ECs of the HFD group (supplementary Fig. 2g). In addition, in the ECs of the HFD group, the endoplasmic reticulum expanded significantly, the mitochondrial cristae disappeared, the mitochondrial density decreased, and the intercellular space increased (supplementary Fig. 2g). However, PEMFs ameliorated ultrastructural damage to ECs (supplementary Fig. 2g). Notably, these benefits occurred independently of body weight changes (supplementary Fig. 2h). PEMFs also improved the serum lipid profile (total cholesterol (TC), total triglyceride (TG), low-density lipoprotein cholesterol (LDL-C), and TG/high-density lipoprotein cholesterol (HDL-C) decreased; HDL-C increased; supplementary Fig. 2i–m) without significantly altering the liver function markers alanine aminotransferase (ALT) and aspartate aminotransferase (AST) (supplementary Fig. 2n, o). Nontargeted metabolomics further indicated that PEMFs modulated glycerophospholipid and cholesterol metabolism (Fig. 1i, j).

To validate the clinical relevance of NLRP3, we performed plasma proteomics in coronary heart disease (CHD) patients and non-CHD controls, which revealed significant enrichment of the NLRP3 inflammasome, IL-18 receptor complex, and pyroptosis pathways in CHD patients (supplementary Fig. 3a, b). Consistent with these findings, an enzyme-linked immunosorbent assay (ELISA) confirmed elevated circulating IL-1β and IL-18 in CHD patients (supplementary Fig. 3c, d).

To identify the specific cellular targets mediating the anti-inflammatory effects of PEMFs, we performed quantitative colocalization analysis of NLRP3 with three major atherosclerotic cell types (ECs (CD31 + ), macrophages (CD68 + ), and vascular smooth muscle cells (α-SMA + )) via Pearson’s correlation coefficient (PCC) and Manders’ coefficient in ImageJ software. The PCC results revealed that NLRP3 was expressed mainly in ECs (PCC = 0.60 ± 0.06) and macrophages (PCC = 0.64 ± 0.05) in plaques, with minimal expression in vascular smooth muscle cells (PCC = 0.30 ± 0.10, supplementary Fig. 2p, q). NLRP3 suppression was more pronounced in ECs than in macrophages following PEMF treatment (Fig. 1k–m, supplementary Fig. 2r). Additionally, PEMFs significantly attenuated endothelial activation, as shown by reduced intercellular adhesion molecule-1 (ICAM-1) expression (supplementary Fig. 2s). These results collectively identify ECs as the primary cellular mediators of PEMFs’ NLRP3-targeted anti-inflammatory action in atherosclerotic plaques.

### PEMFs reduce the oxidized LDL (Ox-LDL)-induced inflammatory response and pyroptosis and improve EC survival in an inflammatory environment

To investigate the effects of PEMFs on EC pyroptosis, we conducted in vitro studies using human umbilical vein endothelial cells (HUVECs) and human aortic endothelial cells (HAECs). First, we explored three PEMF parameter sets in atherosclerosis in vitro models to identify optimal treatment conditions: (1) 5 Hz/0.5 mT/60 min/once (PEMFs I group), (2) 15 Hz/1.5 mT/60 min/once (PEMFs II group), and (3) 30 Hz/3.0 mT/60 min/once (PEMFs III group). The CCK8 assay results revealed that all three PEMF regimens significantly restored Ox-LDL-impaired cell viability, with the most significant improvements observed in the Ox-LDL+PEMFs I group and the Ox-LDL+PEMFs II group (supplementary Fig. 1e). During pyroptosis, pores are formed in the cell membrane, which leads to the release of cell contents, as determined by the lactate dehydrogenase (LDH) release test.^22^ LDH release assays indicated that all three PEMF regimens significantly reduced LDH release. Among them, the improvement observed in the Ox-LDL+PEMFs II group was the most significant (supplementary Fig. 1f). On this basis, we ultimately chose 15 Hz/1.5 mT/60 min/once as the treatment parameter for the subsequent in vitro experiments.

**Fig. 2:**
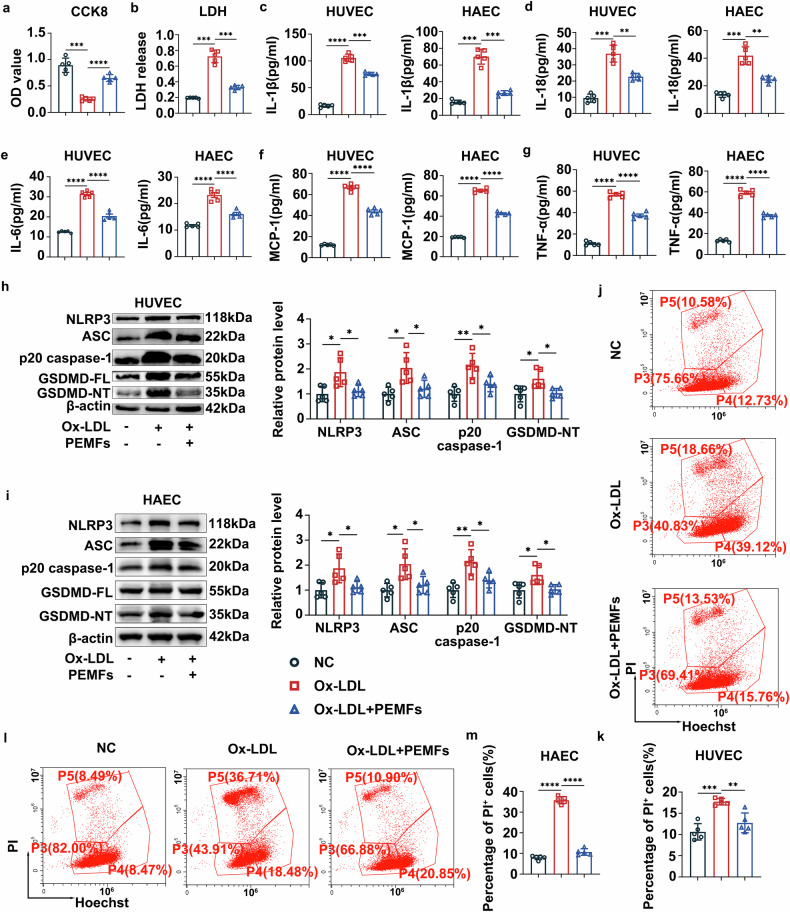
PEMFs reduce the Ox-LDL-induced inflammatory response and pyroptosis and improve EC survival in an inflammatory environment. **a** CCK8 was used to detect the viability of HUVECs in the following groups: the normal cell (NC) group, the Ox-LDL (100 μg/mL, 24 h) group, and the Ox-LDL+PEMFs (15 Hz, 1.5 mT, 1 h/day, 1 d) group; *n* = 5 independent experiments per group. **b** LDH release in the supernatant of HUVECs was detected with an LDH kit; *n* = 5 independent experiments per group. **c**–**g** Levels of IL-1β, IL-18, IL-6, MCP-1, and TNF-α in the supernatants of HUVECs or HAECs were detected via ELISA; *n* = 5 independent experiments per group. **h**, **i** Western blot analysis of NLRP3, ASC, p20 caspase-1, and GSDMD-NT in HUVECs or HAECs; *n* = 5 independent experiments per group. **j**, **k** Pyroptosis was assessed by flow cytometry via Hoechst 33342/PI staining in HUVECs; *n* = 5 independent experiments per group. **l**, **m** Pyroptosis was assessed by flow cytometry via Hoechst 33342/PI staining in HAECs; *n* = 5 independent experiments per group. All the data represent biological replicates. The measured data are presented as the mean ± SEM. Statistical significance was assessed by one-way ANOVA with Tukey’s multiple comparison test. *p < 0.05, **p < 0.01, ***p < 0.001, ****p < 0.0001

Compared with the Ox-LDL group, the Ox-LDL+PEMFs group presented restored cell viability and cell membrane integrity (Fig. 2a, b). Furthermore, PEMFs markedly suppressed the secretion of proinflammatory factors (IL-1β, IL-18, IL-6, MCP-1, and TNF-α) in both HUVECs and HAECs (Fig. 2c–g). WB analysis confirmed that PEMFs downregulated the protein expression of NLRP3, ASC, p20 caspase-1, and GSDMD-NT in both HUVECs and HAECs (Fig. 2h, i). Flow cytometric analysis of PI-positive staining revealed that PEMFs significantly reduced pyroptosis in both cell types (Fig. 2j–m). Collectively, these in vitro findings indicate that PEMFs inhibit Ox-LDL-induced endothelial pyroptosis and inflammation through the suppression of the NLRP3 inflammasome pathway.

### The bidirectional regulation of NLRP3: downregulation has the same effect as that of PEMFs, whereas excessive upregulation counteracts the therapeutic effect of PEMFs

**Fig. 3:**
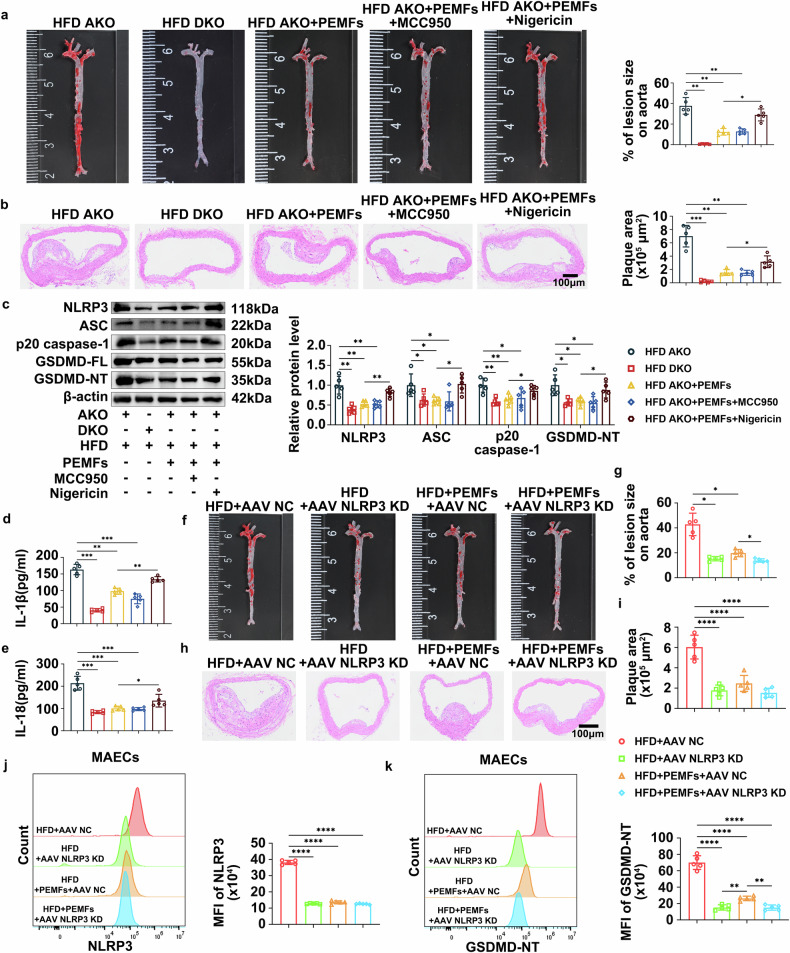
The bidirectional regulation of NLRP3 downregulation has the same effect on PEMFs, whereas excessive upregulation counteracts the therapeutic effect of PEMFs. **a** Oil red O staining was used to determine the lesion burden in the aorta in the ApoE-/- (AKO) and ApoE-/-NLRP3-/- (DKO) mouse groups: the HFD AKO group, HFD DKO group, HFD AKO+PEMFs group, HFD AKO+PEMFs+MCC950 group, and HFD AKO+PEMFs+Nigericin group, *n* = 5 mice per group. **b** HE staining was used to visualize the plaque area in the aortic arch; *n* = 5 mice per group. Scale bar = 100 μm. **c** Western blot analysis of NLRP3, ASC, p20 caspase-1, and GSDMD-NT in aortic tissues; *n* = 5 mice per group. **d**, **e** Serum levels of IL-1β and IL-18 were detected via ELISA; *n* = 5 mice per group. **f** Oil red O staining was used to determine the lesion burden in the aorta in the following groups of AAV-injected ApoE-/- mice: the HFD + AAV NC group, HFD + AAV NLRP3 KD group, HFD+PEMFs+AAV NC group, and HFD+PEMFs+AAV NLRP3 KD group, *n* = 5 mice per group. **g** Statistical results of oil red O staining in mice in each group; *n* *=* 5 mice per group. **h** HE staining was used to visualize the plaque area in the aortic arch in AAV-injected ApoE-/- mice; *n* = 5 mice per group. Scale bar = 100 μm. **i** Statistical results of HE staining in mice in each group; *n* = 5 mice per group. **j** The mean fluorescence intensity (MFI) of NLRP3 was measured via flow cytometry in mouse vascular endothelial cells (MVECs) isolated from heart and aortic tissues; *n* = 5 mice per group. **k** The MFI of GSDMD-NT in MVECs isolated from heart and aortic tissues (*n* = 5 mice per group) was measured via flow cytometry. All the data represent biological replicates. The measured data are presented as the mean ± SEM. Statistical significance was assessed by one-way ANOVA with Tukey’s multiple comparison test. *p < 0.05, **p < 0.01, ***p < 0.001, ****p < 0.0001

To delineate the specific role of NLRP3 in the anti-atherosclerotic effects of PEMFs, we employed NLRP3-/-ApoE-/- double knockout (DKO) mice and pharmacologically modulated NLRP3 activity in ApoE-/- (AKO) mice via the inhibitor MCC950 and the agonist nigericin. Notably, NLRP3 deficiency (DKO) nearly completely abolished plaque formation, indicating that NLRP3 is a key contributor to atherosclerosis (Fig. 3a, b). Conversely, NLRP3 activation via nigericin in AKO mice significantly attenuated the protective effects of PEMFs, increasing the lesion burden, plaque area, and vascular permeability (Fig. 3a, b, supplementary Fig. 4a). In contrast, NLRP3 inhibition with MCC950 phenocopied the benefits of PEMFs, and no additive effect was observed when MCC950 was combined with PEMFs, confirming that both interventions operate through a shared, NLRP3-dependent mechanism. At the molecular level, nigericin upregulated the protein expression of NLRP3 inflammasome components (NLRP3, ASC, and p20 caspase-1) and GSDMD-NT, whereas genetic knockout (DKO) or pharmacological inhibition (MCC950) of NLRP3 downregulated these markers (Fig. 3c). Mirroring these findings, nigericin promoted the secretion of proinflammatory cytokines (IL-1β, IL-18, IL-6, TNF-α, and MCP-1), which was suppressed by NLRP3 ablation or inhibition (Fig. 3d, e, supplementary Fig. 4g–i). Furthermore, nigericin significantly exacerbated dyslipidemia; elevated the serum levels of TG, TC, LDL-C, and TG/HDL-C; and reduced HDL-C (supplementary Fig. 4b–f). Conversely, both genetic and pharmacological NLRP3 inhibition drove lipid normalization (supplementary Fig. 4b–f). Collectively, these results demonstrate that NLRP3 is a critical driver of the pathogenesis of atherosclerosis and that PEMFs confer protection primarily through the inhibition of NLRP3-mediated inflammation and pyroptosis.

### Endothelial-specific NLRP3 knockdown attenuates inflammation and atherosclerotic progression

To determine the endothelial-specific role of NLRP3, we developed an AAV-based knockdown approach (AAV2/9-TIE1p-MCS-mCherry-NLRP3-shRNA) for targeted NLRP3 knockdown in ECs. After three candidates were screened in vivo, the AAV NLRP3 KD-1 construct was selected for subsequent experiments because of its superior transduction efficiency (98.46 ± 1.06% mCherry+ MVECs in AAV NLRP3 KD-1, 96.23 ± 1.32% mCherry+ MVECs in AAV NLRP3 KD-2, 95.98 ± 1.18% mCherry+ MVECs in AAV NLRP3 KD-3, 88.01 ± 4.18% mCherry+ MVECs in AAV NC, supplementary Fig. 5a) and potent NLRP3 knockdown efficacy (supplementary Fig. 5b, c), as confirmed by flow cytometry. Immunofluorescence confirmed the predominant EC-specific localization of the mCherry signal (supplementary Fig. 5d, e). Endothelial-specific NLRP3 knockdown significantly attenuated the atherosclerotic lesion burden and plaque area compared with those of the control (Fig. 3f–i). This effect was associated with reduced NLRP3 and GSDMD-NT expression in isolated MVECs (Fig. 3j, k) and decreased colocalization of NLRP3 with CD31+ ECs (supplementary Fig. 5f, g). Furthermore, endothelial NLRP3 knockdown markedly suppressed the secretion of the proinflammatory factors IL-1β, IL-18, IL-6, MCP-1, and TNF-α (supplementary Fig. 5h–l). Notably, the combination of PEMFs and endothelial NLRP3 knockdown did not yield additive benefits in most readouts, including plaque area, except for a further reduction in IL-6 (Fig. 3f–i, supplementary Fig. 5i). These findings suggest that PEMFs and genetic NLRP3 silencing in ECs converge on a common mechanistic pathway to mitigate atherosclerosis.

### Downregulation of NLRP3 can protect cells, and upregulation of NLRP3 can aggravate EC damage

**Fig. 4:**
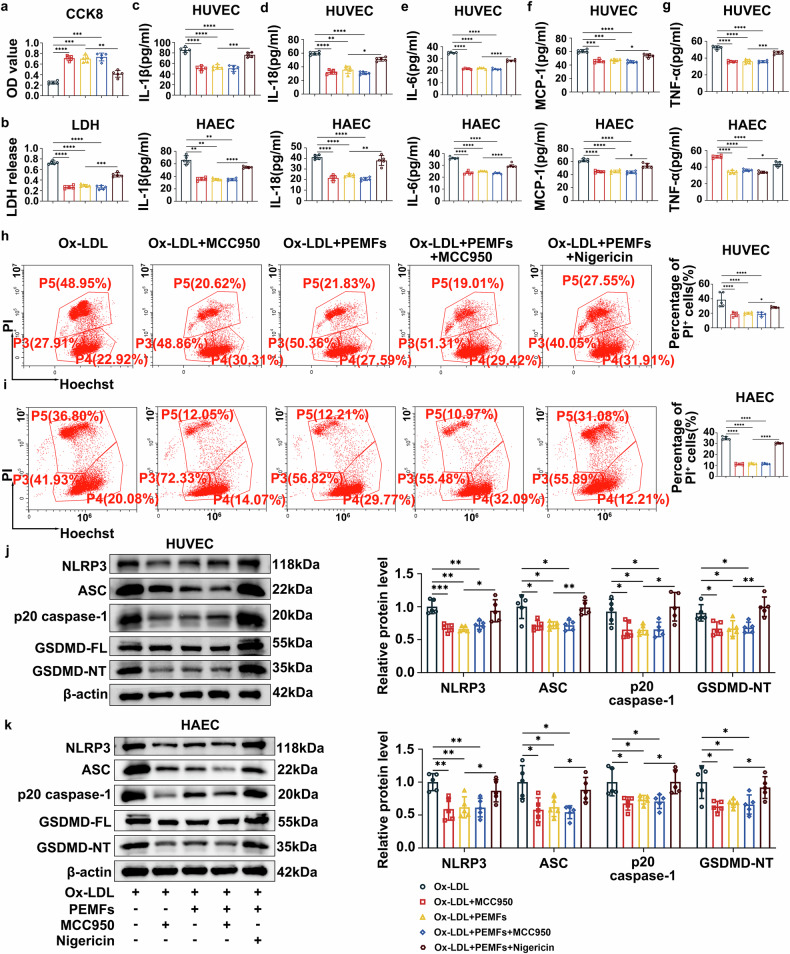
Downregulation of NLRP3 can protect cells, and upregulation of NLRP3 can aggravate EC damage. **a** CCK8 was used to detect cell viability in the following HUVEC groups: the Ox-LDL group, the Ox-LDL + MCC950 group, the Ox-LDL+PEMFs group, the Ox-LDL+ PEMFs+MCC950 group, and the Ox-LDL+PEMFs+Nigericin group; *n* = 5 independent experiments per group. **b** LDH release in the supernatant of HUVECs was detected with an LDH kit; *n* = 5 independent experiments per group. **c**–**g** Levels of IL-1β, IL-18, IL-6, MCP-1, and TNF-α in the supernatants of HUVECs or HAECs were detected via ELISA; *n* = 5 independent experiments per group. **h** Pyroptosis was assessed by flow cytometry via Hoechst 33342/PI staining in HUVECs; *n* = 5 independent experiments per group. **i** Pyroptosis was assessed by flow cytometry via Hoechst 33342/PI staining in HAECs; *n* = 5 independent experiments per group. **j**, **k** Western blot analysis of NLRP3, ASC, p20 caspase-1, and GSDMD-NT in HUVECs or HAECs; *n* = 5 independent experiments per group. All the data represent biological replicates. The measured data are presented as the mean ± SEM. Statistical significance was assessed by one-way ANOVA with Tukey’s multiple comparison test. *p < 0.05, **p < 0.01, ***p < 0.001, ****p < 0.0001

To further delineate the specific role of NLRP3 in mediating the effects of PEMFs, we employed pharmacological modulation via the NLRP3 inhibitor MCC950 and the agonist nigericin in HUVECs and HAECs. The NLRP3 agonist nigericin significantly counteracted the effects of PEMF treatment, as evidenced by reduced cell viability (Fig. 4a), increased LDH release (Fig. 4b), increased secretion of proinflammatory factors (IL-1β, IL-18, IL-6, MCP-1, and TNF-α; Fig. 4c–g), elevated expression of NLRP3 inflammasome components (NLRP3, ASC, and p20 caspase-1; Fig. 4j, k), and increased pyroptosis (increased PI uptake and GSDMD-NT cleavage; Fig. 4h–k). In contrast, the NLRP3 inhibitor MCC950 reproduced the cytoprotective, anti-inflammatory, and anti-pyroptotic effects of PEMFs. No significant differences were observed between the Ox-LDL + MCC950, Ox-LDL+PEMFs, and Ox-LDL+PEMFs+ MCC950 groups across all the measured parameters, indicating that MCC950 and PEMFs are equally effective and nonadditive in terms of protection. These results demonstrate that the beneficial effects of PEMFs are specifically mediated through the inhibition of the NLRP3 inflammasome pathway and its downstream pyroptotic executioner GSDMD.

### Mitochondrial dysfunction is a bridge between inflammation and atherosclerosis

**Fig. 5:**
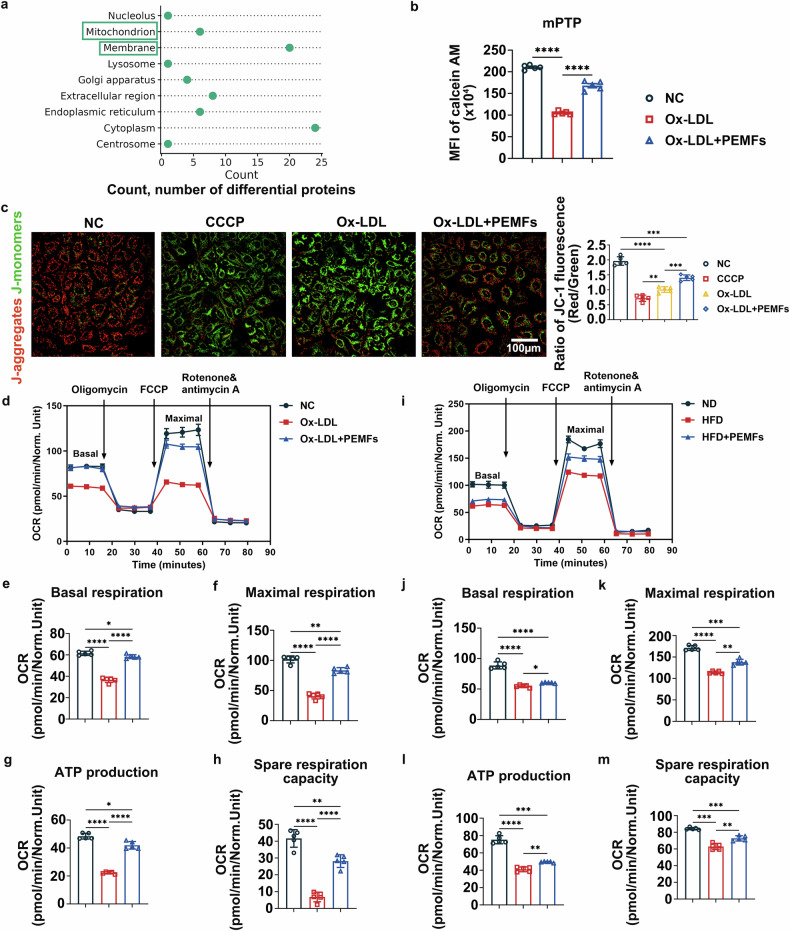
Mitochondrial dysfunction is a bridge between inflammation and atherosclerosis. **a** Subcellular localization analysis of differentially expressed proteins in the CHD group versus the non-CHD group. The count indicates the number of differentially expressed proteins. *n* = 20 participants per group. **b** Mitochondrial permeability transition pore (mPTP) opening was detected with an mPTP detection kit in the following HUVEC groups: the NC group, the Ox-LDL group, and the Ox-LDL+PEMFs group; *n* = 5 independent experiments per group. **c** Mitochondrial membrane potential was detected by JC-1 staining; *n* = 5 independent experiments per group. Scale bar = 100 μm. **d** OCRs of HUVECs subjected to sequential injections of oligomycin (1 mM), FCCP (0.5 mM), and rotenone/antimycin A (0.5 mM). The values were normalized to the cell count. **e**–**h** Bar graphs quantifying basal respiration, maximal respiration, ATP production and spare respiration capacity; *n* = 5 independent experiments per group. **i** OCRs of MVECs subjected to sequential injections of oligomycin (1 mM), FCCP (0.5 mM), and rotenone/antimycin A (0.5 mM) were measured. The values were normalized to the cell count. **j**–**m** Bar graphs quantifying basal respiration, maximal respiration, ATP production and spare respiration capacity; *n* = 5 mice per group. All the data represent biological replicates. The measured data are presented as the mean ± SEM. Statistical significance was assessed by one-way ANOVA with Tukey’s multiple comparison test. *p < 0.05, **p < 0.01, ***p < 0.001, ****p < 0.0001

On the basis of our findings that PEMFs inhibit atherosclerosis via the NLRP3 inflammasome, we next investigated how this external stimulus influences specific intracellular processes. Currently, some studies suggest that the development of atherosclerotic plaques is caused by abnormal local inflammatory reactions of vascular ECs and that this inflammatory response is caused by dysfunction of the cells’ own mitochondria.^23^ Our human proteomic data indicated that mitochondrial and cell membrane alterations were among the most significantly enriched subcellular localizations in CHD patients (Fig. 5a), leading us to hypothesize that mitochondrial dysfunction drives endothelial inflammation. A series of functional assays confirmed that Ox-LDL induced severe mitochondrial dysfunction in HUVECs, which was markedly ameliorated by PEMF treatment. Specifically, PEMFs suppressed Ox-LDL-induced mPTP opening (Fig. 5b), restored the mitochondrial membrane potential (Fig. 5c), and improved mitochondrial respiratory function (basal respiration, maximal respiration, ATP production, and spare respiratory capacity) in HUVECs and MVECs (Fig. 5d–m). Furthermore, PEMFs significantly reduced the Ox-LDL-induced increase in the levels of mitochondrial ROS and cellular superoxide anions (supplementary Fig. 6a, b) and attenuated aberrant calcium release (supplementary Fig. 6c). The abnormal release of Ca^2+^ increases after mitochondrial dysfunction, which can promote the activation of the NLRP3 inflammasome, intensify the inflammatory response, and promote the calcification and instability of atherosclerotic plaques.^24^ We also found that PEMFs enhanced mitophagy, increasing the formation of mitophagosomes both in vitro and in vivo (supplementary Fig. 6d–g), suggesting improved clearance of damaged mitochondria.

In summary, PEMFs ameliorate Ox-LDL-induced mitochondrial dysfunction, reduce oxidative stress and calcium dysregulation, and promote mitophagy, thereby attenuating NLRP3 inflammasome activation and improving endothelial function.

### EC membrane tension and transient receptor potential vanilloid 4 (TRPV4) mechanosensitive channels participate in atherosclerosis-induced endothelial damage

**Fig. 6:**
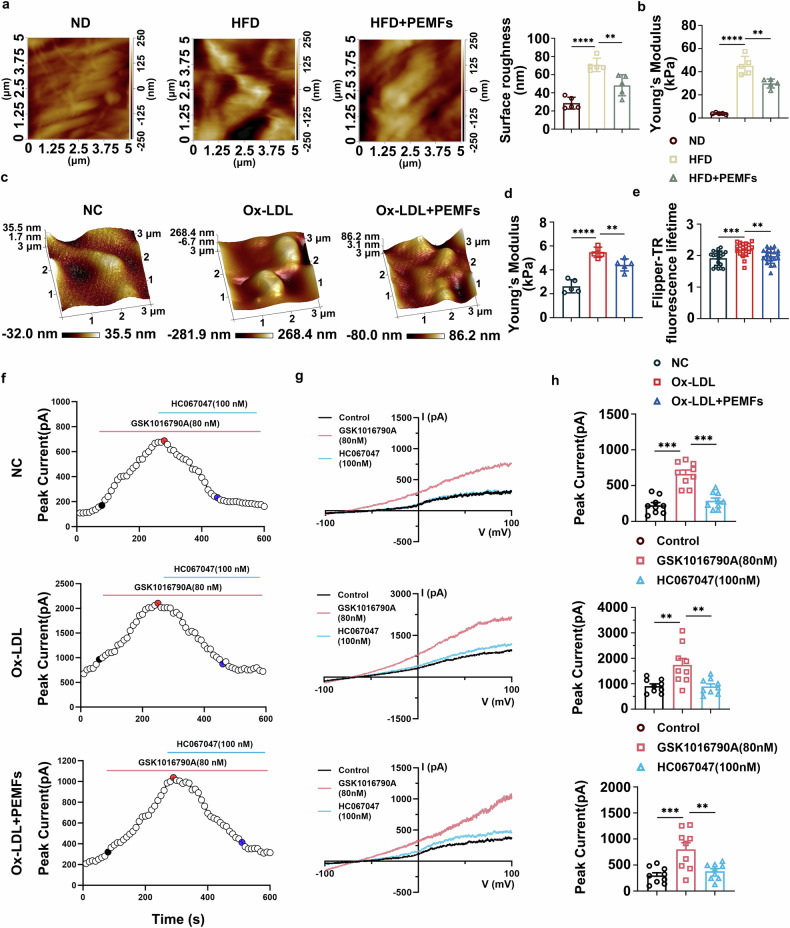
EC membrane tension and transient receptor potential vanilloid 4 (TRPV4) mechanosensitive channels participate in Ox-LDL/HFD-induced endothelial damage. **a** Imaging of the aortic intima of the ND, HFD, and HFD+PEMFs groups in contact mode via atomic force microscopy (AFM). The ultrastructures of fresh intima tissue are depicted in each image. Changes in the surface roughness of vascular ECs in each group; *n* = 5 mice per group. **b** Young’s modulus values of vascular ECs in each group; *n* = 5 mice per group. **c** Topographical images of HUVECs taken with AFM in the following HUVEC groups: the NC group, the Ox-LDL group, and the Ox-LDL+PEMFs group. **d** Young’s modulus values of HUVECs in each group; *n* = 5 independent experiments per group. **e** A Flipper-TR kit was used to observe cell membrane tension; *n* = 20 cells per group. **f** Time course of HUVEC membrane currents measured at baseline and after the application of GSK1016790A (80 nM) and HC067047 (100 nM). The ramp pulses were applied every 10 s from −100 to +100 mV for 1 s from a holding potential of 0 mV. **g** Representative current–voltage curves taken at the three time points corresponding to the black, red, and blue dots in (**a**). **h** Peak current amplitudes measured at +100 mV under baseline conditions (control), after GSK1016790A application and after HC067047 application. Each dot represents the peak current amplitude measured from one HUVEC; *n* = 9 cells per group. All the data represent biological replicates. The measured data are presented as the mean ± SEM. Statistical significance was assessed by one-way ANOVA with Tukey’s multiple comparison test. *p < 0.05, **p < 0.01, ***p < 0.001, ****p < 0.0001

Proteomic analysis highlighted alterations in the cell membrane as a key feature in atherosclerosis (Fig. 5a), prompting us to investigate whether membrane structural changes and mechanosensory pathways link external stimuli to intracellular signaling. We used atomic force microscopy (AFM) to perform mechanical and morphological imaging of the aortic intima. The morphological images revealed that the ECs in the ND group were oval or long spindle shaped, with a close and regular arrangement. In contrast, ECs in the HFD group were swollen and deformed, arranged in a disorderly manner, and formed ridge-like structures. In the HFD+PEMFs group, the cell morphology gradually recovered to a long spindle shape, the arrangement tended to be regular, and the number of ridge-like structures decreased (Fig. 6a). A comparison of the surface roughness of the aortic intima revealed that the roughness of the HFD group was significantly greater than that of the other two groups (Fig. 6a). Additionally, Young’s modulus data indicated that a HFD significantly increased aortic stiffness, whereas PEMFs alleviated this effect (Fig. 6b, supplementary Fig. 7a). AFM also revealed that Ox-LDL induced pathological morphological changes in HUVECs, including increased cell height, a reduced spreading area, and increased cellular stiffness. PEMF treatment effectively ameliorated these alterations, promoting cell spreading and reducing stiffness (Fig. 6c, d, supplementary Fig. 7b, c). Consistent with these findings, fluorescence imaging via a membrane tension probe (Flipper-TR) revealed that Ox-LDL significantly increased plasma membrane tension, which was attenuated by PEMFs (Fig. 6e). Phalloidin staining of F-actin further demonstrated that F-actin clustered in the peripheral region in the Ox-LDL group, with individual stress fibers partially disappearing, cells contracting, and intercellular gaps widening, indicating redistribution of F-actin. In the presence of PEMFs, the stress fibers gradually extended, and the cell morphology became regular (supplementary Fig. 7d). We next examined the role of mechanosensitive channels. WB analysis revealed that TRPV4 was consistently upregulated under pro-atherogenic conditions in Ox-LDL-treated HUVECs and HAECs, as well as in the aortic tissues of HFD-fed ApoE-/- mice. PEMF intervention significantly reversed this upregulation (supplementary Fig. 7e–g). In contrast, the expression of transient receptor potential canonical 1 (TRPC1) remained unchanged between the Ox-LDL/HFD groups and the Ox-LDL+PEMFs/ HFD+PEMFs groups (supplementary Fig. 7h–j). To further confirm the role of TRPV4 in ECs, we first performed patch‒clamp assays on HUVECs to evaluate the effects of disease state and PEMF treatment on TRPV4 channel activity. During voltage ramp stimulation (±100 mV), the TRPV4 agonist GSK1016790A evoked both inward and outward transmembrane currents, which were effectively blocked by the antagonist HC067047 (Fig. 6f–h). Compared with those in the NC group, HUVECs in the Ox-LDL group presented a greater baseline current level, a more sensitive agonist response, and increased peak currents. In contrast, the Ox-LDL+PEMFs group presented significantly lower baseline levels and peak currents than did the Ox-LDL group (Fig. 6f–h). These results indicated that Ox-LDL upregulated TRPV4 protein expression and enhanced the sensitivity of TRPV4 channels to the agonist GSK1016790A. We subsequently conducted quantitative colocalization analysis of TRPV4 and CD31 via the PCC and Manders’ coefficients. The results revealed that TRPV4 and CD31 were colocalized and that the expression of TRPV4 in ECs was inhibited after PEMF treatment (supplementary Fig. 7k–m). These results identify TRPV4 as the key mechanosensor mediating the protective effects of PEMFs.

### TRPV4 hyperactivation exacerbates cellular stiffness, mitochondrial dysfunction, and inflammation, whereas TRPV4 inhibition confers cytoprotection

**Fig. 7:**
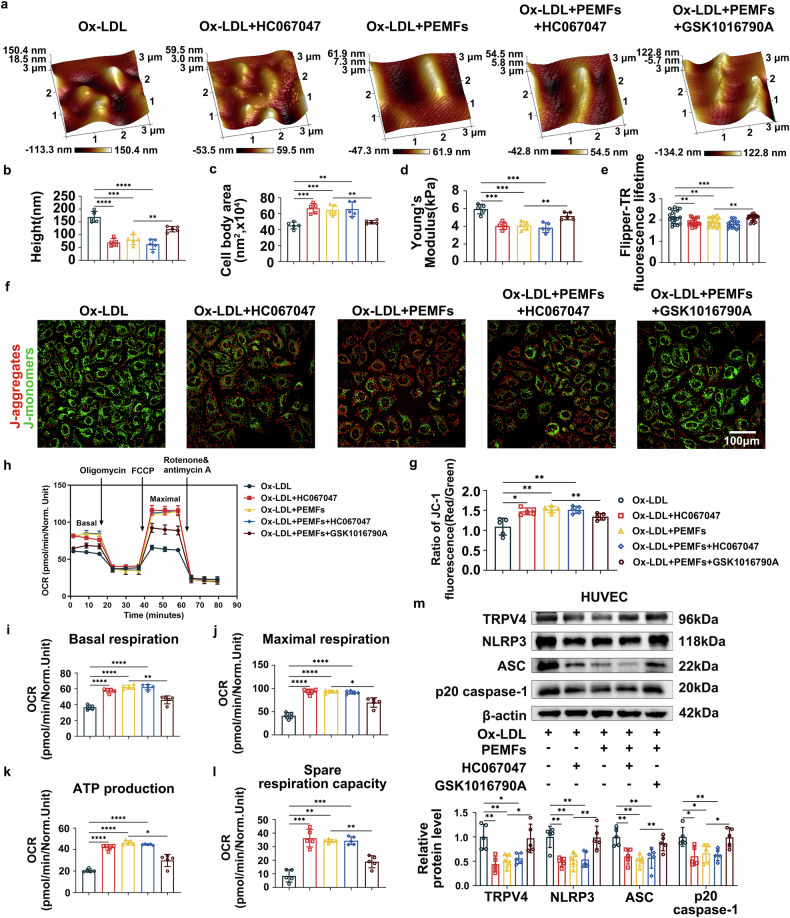
TRPV4 hyperactivation exacerbates cellular stiffness and mitochondrial dysfunction, whereas TRPV4 inhibition confers cytoprotection. **a** Topographical images of HUVECs from the following HUVEC groups: Ox-LDL group, Ox-LDL + HC067047 group, Ox-LDL+PEMFs group, Ox-LDL+PEMFs+HC067047 group, and Ox-LDL+PEMFs+GSK1016790A group. **b**, **c** Cell height and cell body area were calculated from topographical images of HUVECs; *n* = 5 independent experiments per group. **d** Young’s modulus values of HUVECs in each group; *n* = 5 independent experiments per group. **e** A Flipper-TR kit was used to observe cell membrane tension; *n* = 20 cells per group. **f**, **g** Mitochondrial membrane potential was detected by JC-1 staining; *n* = 5 independent experiments per group. Scale bar = 100 μm. **h** OCRs of HUVECs subjected to sequential injections of oligomycin (1 mM), FCCP (0.5 mM), and rotenone/antimycin A (0.5 mM) were measured. The values were normalized to the cell count. **i**–**l** Bar graphs quantifying basal respiration, maximal respiration, ATP production, and spare respiratory capacity; *n* = 5 independent experiments per group. **m** Western blot analysis of TRPV4, NLRP3, ASC, and p20 caspase-1 protein expression in HUEVCs; *n* = 5 independent experiments per group. All the data represent biological replicates. The measured data are presented as the mean ± SEM. Statistical significance was assessed by one-way ANOVA with Tukey’s multiple comparison test. *p < 0.05, **p < 0.01, ***p < 0.001, ****p < 0.0001

To define the role of TRPV4 in PEMF-mediated protection, we pharmacologically modulated its activity via the agonist GSK1016790A and the inhibitor HC067047. TRPV4 activation with GSK1016790A effectively abolished the beneficial effects of PEMFs. It impaired cell viability (supplementary Fig. 8a), increased LDH release (supplementary Fig. 8b), and enhanced the secretion of proinflammatory factors (IL-1β, IL-6, IL-18, MCP-1, and TNF-α; supplementary Fig. 8c-l) in both HUVECs and HAECs. Furthermore, it induced cellular contraction (Fig. 7a–c), increased membrane stiffness and tension (Fig. 7d, e), peripheral F-actin condensation (supplementary Fig. 9a), and severely compromised mitochondrial function, including reducing membrane potential (Fig. 7f, g), increasing ROS production (supplementary Fig. 9b, c, e), calcium ion influx (supplementary Fig. 9d), and mPTP opening (supplementary Fig. 9f), impairing mitophagy (supplementary Fig. 9g), and diminishing the OCR across all key parameters (basal respiration, maximal respiration, ATP production, spare capacity, Fig. 7h–l). Consequently, it also upregulated the protein expression of the NLRP3 inflammasome and its components (Fig. 7m, supplementary Fig. 9h). Conversely, TRPV4 inhibition with HC067047 perfectly recapitulated the cytoprotective, anti-inflammatory, mechanoprotective, and mitochondrial-stabilizing effects of PEMFs. No significant differences were observed between the Ox-LDL + HC067047, Ox-LDL+PEMFs, and Ox-LDL+PEMFs+HC067047 groups across all the assays. These results demonstrate that TRPV4 is the critical mechanosensitive target through which PEMFs exert their therapeutic effects and that its inhibition is both necessary and sufficient to mediate protection against Ox-LDL-induced endothelial damage.

### TRPV4 activation exacerbates atherosclerosis, whereas TRPV4 inhibition has the same effect on PEMFs

**Fig. 8:**
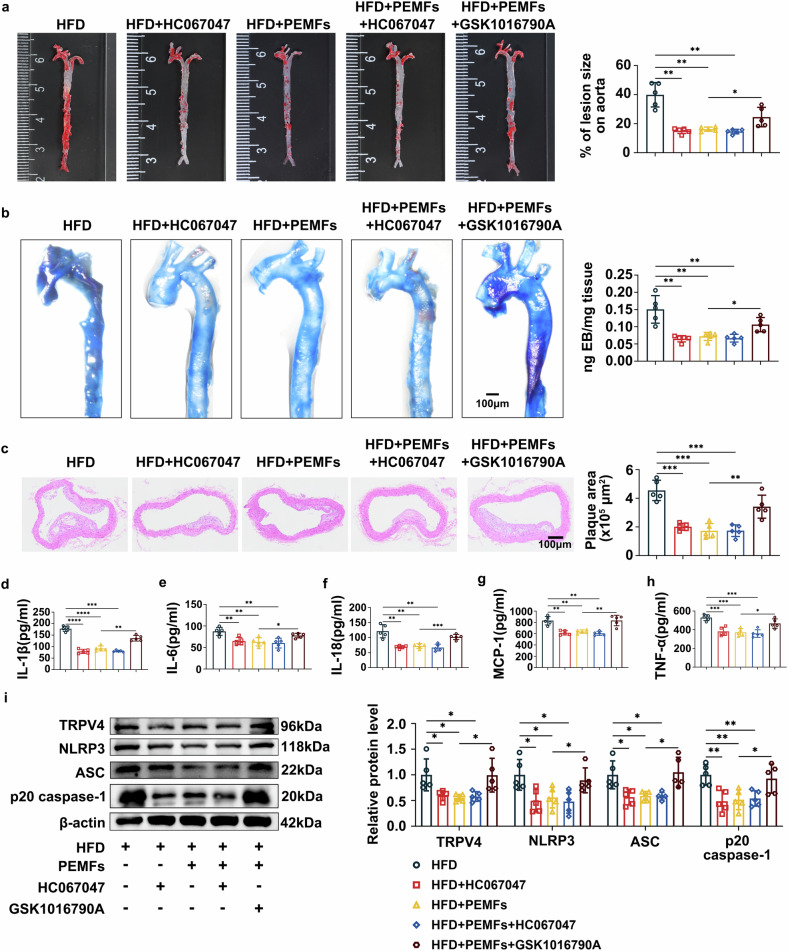
TRPV4 activation exacerbates atherogenesis, whereas TRPV4 inhibition has the same effect on PEMFs. **a** Oil red O staining was used to determine the lesion burden in the aortas of the ApoE-/- mice in the following groups: the HFD group, HFD + HC067047 group, HFD+PEMFs group, HFD+PEMFs+HC067047 group, and HFD+PEMFs+ GSK1016790A group; *n* = 5 mice per group. **b** Evans blue staining was used to assess aortic permeability in ApoE-/- mice; *n* = 5 mice per group. Scale bar = 100 μm. **c** HE staining was used to visualize the plaque area in the aortic arch. *n* = 5 mice per group. Scale bar = 100 μm. **d**–**h** Serum levels of IL-1β, IL-6, IL-18, MCP-1, and TNF-α were detected by ELISA; *n* = 5 mice per group. **i** Western blot analysis of TRPV4, NLRP3, ASC, and p20 caspase-1 in aortic tissues; *n* = 5 mice per group. All the data represent biological replicates. The measured data are presented as the mean ± SEM. Statistical significance was assessed by one-way ANOVA with Tukey’s multiple comparison test. *p < 0.05, **p < 0.01, ***p < 0.001, ****p < 0.0001

To validate the role of TRPV4 in vivo, we administered HC067047 and GSK1016790A to ApoE-/- mice. GSK1016790A effectively abolished the protective effects of PEMFs, increasing the atherosclerotic lesion burden, plaque area, and vascular permeability (Fig. 8a–c). It also reversed the PEMF-mediated suppression of proinflammatory factors (IL-1β, IL-6, IL-18, MCP-1, and TNF-α; Fig. 8d–h) and upregulated the protein expression of TRPV4 and NLRP3 inflammasome components (Fig. 8i). In contrast, HC067047 phenocopied the beneficial effects of PEMFs, reducing plaque formation, vascular leakage, and inflammation. No additive effect was observed when HC067047 was combined with PEMFs, indicating that they operate through the same pathway. These in vivo results demonstrate that TRPV4 hyperactivation exacerbates atherosclerosis by promoting inflammation and NLRP3 activation, whereas its inhibition confers strong protection, establishing TRPV4 as a key mechanistic target for treatment.

## Discussion

Given the significant clinical burden of atherosclerosis and the limitations of current treatments, noninvasive therapeutic alternatives are urgently needed. PEMFs, although traditionally applied in osteoarthritis, have shown anti-inflammatory effects in conditions such as osteoarthritis and rheumatoid arthritis.^14,15^ On the basis of our previous clinical findings that inflammation is the primary mechanism underlying the therapeutic benefits of PEMFs in knee joints, we extended this approach to cardiovascular diseases. While preliminary clinical evidence indicates that PEMFs can reduce blood pressure, improve vasodilation in hypertensive and aging populations,^20,25^ and increase peripheral circulation in diabetic patients,^26^ research on their vascular applications remains limited. Given the critical role of inflammation in atherosclerosis, we evaluated the effect of PEMFs on its progression. Our study revealed that PEMFs attenuate atherosclerotic plaque progression by inhibiting NLRP3 inflammasome-mediated endothelial pyroptosis and restoring mitochondrial function. These effects were linked to improved membrane tension and downregulation of TRPV4 mechanosensitive channels. In summary, our study identifies PEMFs as a promising noninvasive and nonpharmacological strategy for atherosclerosis intervention, although further translation from preclinical studies is needed.

Our previous work focused on inflammation-related proteins. Coincidentally, one of the central contributors to our findings is the NLRP3 inflammasome, a known linchpin connecting metabolic stress to vascular inflammation.^27,28^ Using systemic NLRP3 knockout in ApoE-/-NLRP3-/- mice and endothelial-specific NLRP3 knockdown models, we confirmed the role of NLRP3 in driving endothelial pyroptosis and the progression of atherosclerosis. Consistent with these findings, the levels of the NLRP3 inflammasome complex were significantly elevated in patients with CHD compared with controls. These findings align with those of previous reports by Zhuang T et al.^29^ who collectively established endothelial NLRP3 as a pivotal driver of atherosclerosis. Further bidirectional pharmacological interventions confirmed that NLRP3 activation abolished the atheroprotective effects of PEMFs, whereas NLRP3 inhibition recapitulated the benefits of PEMFs. Critically, no additive effect was observed when PEMFs were combined with NLRP3 inhibition, indicating that PEMFs act primarily through this pathway. Together, our genetic and pharmacological evidence underscores EC-targeted anti-inflammatory strategies as promising directions for precise atherosclerosis therapy.

Mitochondria serve as central platforms for NLRP3 inflammasome activation, facilitating its assembly and amplifying inflammatory signals such as mtROS.^30^ Integrated proteomic analysis of plasma from patients with CHD and preclinical atherosclerotic models revealed pronounced mitochondrial dysfunction as a hallmark of atherosclerosis. We observed striking structural lesions (swollen/shrunken organelles, crista disorganization) and functional impairments (diminished mitophagy, elevated mtROS levels, compromised respiration) in EC mitochondria. Given the established role of mitochondria as platforms for NLRP3 inflammasome assembly, they provide a bridge for PEMFs to exert anti-inflammatory effects. While PEMFs have been reported to modulate mitochondrial function in musculoskeletal disorders and endothelial angiogenesis,^31,32^ their role in atherosclerosis-related mitochondrial dysfunction remains uncharacterized. Our study fills this gap: Seahorse assays confirmed rescued mitochondrial respiratory function in PEMF-treated HUVECs and MVECs compared with that in atherosclerotic controls. In vitro, PEMFs also restored the mitochondrial membrane potential, reduced the ROS and Ca^2+^ levels, and increased mitophagy. In vivo, PEMFs significantly improved aortic endothelial mitochondrial integrity (restored membrane structure, matrix density, and cristae organization). Collectively, these findings demonstrated that PEMFs mitigate atherosclerosis by reversing mitochondrial structural and functional deficits (including impaired respiration), thereby inhibiting NLRP3 inflammasome activation and endothelial pyroptosis.

The mechanisms through which external electromagnetic fields influence intracellular organelles remain incompletely understood. Stephenson et al. reported that PEMFs modulated membrane dynamics (reduced phosphodiester/ATP ratios), suggesting an effect on membrane tension.^31^ Our integrated proteomic and functional analyses revealed that PEMFs preferentially target membrane-associated proteins, supporting the hypothesis that PEMFs act through mechanical modulation. Importantly, AFM further revealed structural and mechanical perturbations in atherosclerotic ECs. Compared with control cells, atherosclerotic cells presented height elevation, cell contraction, and an increased elastic modulus, which was consistent with the increase in membrane tension reflected by Flipper-TR. These mechanical abnormalities were reversed by PEMFs, suggesting that PEMFs improve endothelial biomechanics.

We next explored potential mechanosensitive mediators. Another study linked brief PEMF exposure to enhanced myogenesis and metabolic balance through the regulation of the TRPC1 channel,^33^ a TRP family member involved in myoblast fusion and immune regulation. Although TRPC1 has been studied in atherosclerosis, most studies have focused on its role in vascular smooth muscle cells rather than ECs.^34^ One study also revealed that TRPC1 has strong fluorescent signals in macrophages in plaques and weak signals in the intimal layer.^35^ Notably, our WB analyses also revealed no significant changes in TRPC1 expression in the atherosclerosis or PEMF groups, ruling out its involvement. In contrast, TRPV4, another TRP family member expressed in ECs, is linked to osmotic, thermal, and mechanical sensing.^36^ On the basis of its characteristics, TRPV4 may be a more suitable target than TRPC1 for treating atherosclerosis in PEMFs. TRPV4 expression was markedly upregulated in atherosclerotic mice and Ox-LDL ECs, whereas its levels were normalized in PEMFs. More importantly, the patch-clamp results demonstrated that Ox-LDL not only increased TRPV4 expression but also increased TRPV4 sensitivity to GSK1016790A, while PEMFs attenuated these effects. This specificity (TRPV4 but not TRPC1) supports TRPV4 as the primary mechanosensitive target of PEMFs in AS.

The role of TRPV4 in atherosclerosis is further supported by its functional links to endothelial dysfunction: shear stress enhances endothelial TRPV4 agonist sensitivity,^37^ and its activation increases ATP release, ROS production, and mitochondrial dysfunction.^38,39^ Our results were consistent with these studies, as elevated mitochondrial oxidative stress, reduced membrane potential, impaired autophagy, decreased respiratory function, and diminished ATP production were observed in ECs overexpressing TRPV4. Notably, TRPV4-mediated mechanical sensing intersects with the AFM-observed biomechanical changes: TRPV4 upregulation in atherosclerosis likely exacerbates membrane tension and disrupts EC mechanical properties (height, area, elastic modulus), whereas TRPV4 downregulation restores these parameters. While TRPV4 has been implicated in macrophage foam cell formation in atherosclerosis,^40,41^ its role in EC inflammation remains understudied. One study revealed that in lung ischemia and reperfusion injury, the loss of EC TRPV4 can effectively improve the partial pressure of blood oxygen and inflammation.^42^ Our study is the first to demonstrate that PEMFs regulate EC mitochondrial function and NLRP3 inflammasome activation through TRPV4, thereby slowing the progression of atherosclerosis. Future work will focus on identifying PEMF binding sites on the six-transmembrane domain of TRPV4, a critical step toward precision therapy, given its complex structure and mechanosensitive properties.

Collectively, our findings demonstrated that PEMFs hold significant promise as a nonpharmacological, noninvasive therapeutic strategy for atherosclerosis. Mechanistically, we revealed that the anti-inflammatory and anti-atherosclerotic effects of PEMFs are mediated through a novel regulatory axis: PEMFs first modulate EC membrane tension to downregulate the mechanosensitive TRPV4 channel, which repairs mitochondrial dysfunction, thereby inhibiting NLRP3 inflammasome activation and subsequent GSDMD-dependent pyroptosis. This cascade collectively mitigated endothelial injury and attenuated the progression of atherosclerosis.

This study has several limitations. First, this study didn’t systematically screen other mechanosensitive molecules, such as members of the TRP family. In subsequent studies, high-throughput approaches will be needed to further clarify the roles of various mechanosensitive molecules. Second, the duration of PEMF intervention in this study was 3 weeks. However, atherosclerosis is a chronic progressive disease that typically develops over years or even decades in humans; thus, this intervention duration cannot fully demonstrate the long-term effects of PEMFs on plaque regression and complication prevention. Third, the human plasma proteomic analysis was based on a small cohort (*n* = 20), and the limited sample size may prevent the generalization of the relevant proteomic findings to a broader population of atherosclerotic patients. Future studies should explore additional optimal parameter combinations through additional preclinical research and conduct clinical trials to verify the safety and efficacy of optimized parameters in atherosclerotic patients, which represents a core step in advancing PEMFs from preclinical research to clinical application.

## Materials and methods

### Clinical sample experimental design

The inclusion criteria for CHD were as follows: (1) CHD diagnosis on the basis of usual angina symptoms and verified by coronary angiography (>50% stenosis in at least 1 major epicardial artery); (2) age over 30 years; and (3) willingness to provide a peripheral blood sample for research analysis. Patients with severe liver or renal illness, any type of malignancy, or severe autoimmune disease (e.g., systemic lupus erythematosus, inflammatory bowel disease, or Graves’ disease) and those who were pregnant or breastfeeding were excluded. Twenty consecutive patients with CHD (CHD group) were enrolled in this study. In addition, 20 additional control participants (non-CHD group) were selected from patients who had undergone coronary angiography to rule out CHD. CHD patients and non-CHD patients were recruited from West China Hospital of Sichuan University. The baseline characteristics of the study population are presented in supplementary Table 1. This trial was approved by the Chinese Clinical Trial Registry (Registration number: ChiCTR2400092082). The principles outlined in the Declaration of Helsinki were followed. Peripheral blood samples were collected from all participants via vacuum blood collection tubes (anticoagulant tube/EDTA K_2_2H_2_O) and centrifuged in a cold centrifuge at 4000 rpm for 10 min. Afterward, the collected plasma was aliquoted into several 1.5 mL Eppendorf tubes (EPs) and stored at −80 °C until further analysis.

### Data independent acquisition (DIA) quantitative proteomics

Human plasma samples were subjected to DIA mass spectrometry (MS). Briefly, plasma proteins were extracted and enzymatically digested into peptides via trypsin. An aliquot of peptides from each sample was subjected to chromatographic separation via a Vanquish Neo UHPLC system (Thermo, USA), followed by DIA MS analysis on an Orbitrap Astral mass spectrometer (Thermo, USA). All MS raw files were processed via DIA-NN software (v1.8) for spectral library search and quantitative proteomic analysis against the UniProtKB *Homo sapiens* (human) protein sequence database. Proteomic analysis was performed by Shanghai Bioprofile Technology.

### AAV-shRNA design and screening

AAV-mediated vascular EC-specific NLRP3-knockdown constructs were constructed by Shanghai OBiO Technology Co., Ltd. (Shanghai, China). Three vascular endothelial-specific NLRP3-targeting shRNA sequences were designed via two independent algorithms: the Thermo Fisher RNAi Designer (https://rnaidesigner.thermofisher.com/rnaiexpress/setOption.do?designOption=sirna&pid=6141821818688777179) and the Broad Institute’s GPP Portal (https://portals.broadinstitute.org/gpp/public/gene/search). The three candidates were as follows: (1) Y40837, CCAGGAGAGAACCTCTTATTT (AAV NLRP3 KD-1 group); (2) Y40838, GGATGAACGTGTTCCAGAAGG (AAV NLRP3 KD-2 group); and 3) Y40839, GCTGGAATCTCTCCACAATTC (AAV NLRP3 KD-3 group). All the AAV constructs were engineered to express the mCherry fluorescent reporter (Ex/Em: 587/610 nm).

Primary mouse vascular endothelial cells (MVECs) were isolated from ApoE-/-mice via CD31+ magnetic-activated cell sorting (Miltenyi Biotec, 130--097--418) and subsequently transduced with either AAV NC, AAV NLRP3 KD-1, AAV NLRP3 KD-2, AAV NLRP3 KD-3, or the PBS vehicle control (untransduced, native group). At 72 h posttransduction, the cells were harvested for flow cytometric analysis to quantify the following: (1) transduction efficiency: percentage of mCherry+ cells; (2) knockdown efficacy: NLRP3 mean fluorescence intensity (MFI) using a goat anti-mouse IgG-Alexa Fluor 488-conjugated antibody (Jackson, 111-545-003, USA; 1:500).

### Extraction of mouse vascular endothelial cells (MVECs)

For the isolation of primary MVECs, mice in the HFD + AAV NC group, HFD + AAV NLRP3 KD group, HFD+PEMFs+AAV NC group, and HFD+PEMFs+AAV NLRP3 KD group were used. Briefly, cells were collected from type II collagenase-digested mouse heart and aorta tissues and enriched with CD31 mouse antibody (BD, 558736, USA)-coated Dynabeads (Invitrogen, 11035, USA). The cells were cultured in DMEM containing 15% FBS with penicillin‒streptomycin, EC supplement (Sigma, B211–GS, USA), and heparin. Primary MVECs were used between passages two and four for the assays.

### Experimental model animal and animal grouping

ApoE-/- mice were purchased from Beijing Huafukang Biotechnology Co., Ltd. Global NLRP3 knockout (NLRP3-/-) mice were kindly donated by Prof. Yubin Luo of Sichuan University. NLRP3-/- mice were crossed with ApoE-/- mice to generate ApoE-/-NLRP3-/- (DKO) mice. Knockout was confirmed by genotyping.

Six-week-old male experimental mice were fed a high-fat diet (HFD) containing 40% fat and 1.25% cholesterol (Xietong Pharmaceutical Bioengineering Co., Ltd., D12108C, China) for twelve weeks. Standard housing conditions were maintained at a controlled temperature (18–23 °C) and humidity (40–60%) with a 12-h light–dark cycle. The animal experimental procedures were reviewed and approved by the Institutional Animal Care and Use Committees of West China Hospital, Sichuan University (Registration numbers: 20211490A).

ApoE-/- mice were randomly assigned to the control group or were treated with a HFD, HFD+PEMFs, HFD+PEMFs+MCC950, HFD+PEMFs+Nigericin, HFD + HC067047, HFD+PEMFs+HC067047, HFD+PEMFs+GSK1016790A, HFD + AAV NC, HFD + AAV NLRP3 KD, HFD+PEMFs+AAV NC, or HFD+PEMFs+AAV NLRP3 KD. Twelve weeks after HFD induction, atherosclerotic mice were injected with MCC950 (10 mg/kg, every 48 h, 3 weeks, tail vein injection; MCE, HY-12815, USA), nigericin (5 mg/kg, every 48 h, 3 weeks, tail vein injection; MCE, HY-127019, USA), HC067047 (10 mg/kg, every 48 h, 3 weeks, tail vein injection; MCE, HY-100208, USA), or GSK1016790A (10 mg/kg, every 48 h, 3 weeks, tail vein injection; MCE, HY-19608, USA). The following sequences were used in the AAV NC groups and the AAV NLRP3 KD groups: AAV2/9-TIE1p-MCS-mCherry-NLRP3, 5′-GGATGAACGTGTTCCAGAAGG-3′; AAV2/9-TIE1p-MCS-mCherry-NC, 5′-GAAGTCGTGAGAAGTAGAA-3′. The titers of AAV2/9-TIE1p-MCS-mCherry-NLRP3 and AAV2/9-TIE1p-MCS-mCherry-NC were 1.67 × 10^13^ (v.g./ml) and 1.48 × 10^13^ (v.g./ml), respectively. AAV2/9-TIE1p-MCS-mCherry-NLRP3 or AAV2/9-TIE1p-MCS-mCherry-NC (5 × 10^12 ^v.g./ml/individual, 100 µL) was injected into the jugular vein 4 weeks before HFD.

### Experimental model cell and cell grouping

The human umbilical vein endothelial cell line (CRL-1730) was purchased from the American Type Culture Collection and cultured with Ham’s F12k (Gibco, 21127022, USA) and 10% fetal bovine serum (FBS, Gibco, 26010074, USA) supplemented with 1% penicillin‒streptomycin (HyClone, SV30010, USA). HUVECs were cultured in a cell incubator at 37 °C and 5% CO_2_. All experiments were conducted using cells at passages 4–10. The HAEC cell line (IM-H359) was purchased from Xiamen Immocell Biotechnology Co., Ltd. (Fujian, China) and cultured in HAEC-specific medium (Immocell, IM-H359-1, China). HAECs were cultured in a cell incubator at 37 °C and 5% CO_2_. All experiments were conducted using cells at passages 4–10.

HUVECs were treated with Ham’s F12k medium and 10% FBS containing 100 μg/mL Ox-LDL (Yiyuan Biotech, YB-002, China) for 24 h to induce the atherosclerotic model, and HAECs were treated with special medium containing 100 μg/mL Ox-LDL for 24 h to induce the atherosclerotic model,^43^ which was referred to as the model group. The control group was left untreated. The cells were cultured in a constant-temperature incubator with 5% CO_2_ at 37 °C.

HUVECs or HAECs were randomly assigned to the control group or treated with Ox-LDL, Ox-LDL+PEMFs, Ox-LDL + MCC950, Ox-LDL+PEMFs+MCC950, Ox-LDL+ PEMFs+Nigericin, Ox-LDL + HC067047, Ox-LDL+PEMFs+HC067047, or Ox-LDL+ PEMFs+GSK1016790A. At 24 h after Ox-LDL induction, HUVECs or HAECs were treated with MCC950 (10 µM, 2 h; MCE, HY-12815, USA), nigericin (5 µM, 2 h; MCE, HY-127019, USA), HC067047 (1 µM, 2 h; MCE, HY-100208, USA), or GSK1016790A (100 nM, 2 h; MCE, HY-19608, USA).

### PEMF intervention in vivo and in vitro

The magnetic field device was manufactured by Sichuan University (Fig. S10).^44^ The mice in the PEMF group were exposed to PEMFs for 3 weeks after 12 weeks of HFD. PEMFs were generated with the following parameters: adjustable pulse wave, frequency = 15 Hz, intensity = 1.5 mT, pulse duration = 0.3 s, pulse duty ratio: 30%, and exposure time = 60 min/day. The parameters of PEMFs in vitro and in vivo follow the experimental results of previous studies and the parameter gradients used in this study.^45,46^ The parameters of the PEMFs were divided into three groups for the experiments to screen out the optimal parameters (1.5 Hz/0.5 mT/60 min/3 weeks (PEMF I group), 2.15 Hz/1.5 mT/60 min/3 weeks (PEMF II group), 3. 30 Hz/3.0 mT/60 min/3 weeks (PEMF III group)). For in vivo experiments, the main focuses were on lesion burden (Oil red O staining) and the secretion of proinflammatory factors (ELISA). For in vitro experiments, the main focuses were on cell viability (CCK8 assay) and cell membrane permeability (LDH assay). The mice were placed in the PEMF instrument for intervention. The mice in the atherosclerotic control group received sham PEMFs for the same amount of time without any output. The in vitro PEMF intervention device was installed in the cell incubator. HUVECs were exposed to PEMFs, and the frequency and intensity (15 Hz, 1.5 mT) were the same as those used for PEMF intervention in vivo.

### Plasma lipid biochemical analysis

After PEMF intervention, the mice were anesthetized, and blood samples were collected. Serum lipid profiles, including TC, TG, LDL-C, and HDL-C, were measured via enzymatic assay with an automatic biochemical analyzer (Thermo Scientific, MGC240, USA).

The liver function indicators AST and ALT were detected via activity assay kits (Solarbio, BC6435, BC1550, China). The corresponding standard solutions were prepared according to the manufacturer’s instructions. The standards and serum samples to be tested were added to 96-well plates. After the reaction was completed, the absorbance values were measured via a microplate reader, and the activity values of AST and ALT were calculated according to predetermined formulas.

### Enzyme-linked immunosorbent assay (ELISA)

Plasma samples were obtained from blood samples collected from clinical patients and mice, and supernatants were obtained from HUVECs and HAECs of each group. Mouse IL-1β (Ruixinbio, RX203063M, China), mouse IL-18 (Ruixinbio, RX203064M, China), mouse IL-6 (Ruixinbio, RX203049M, China), mouse MCP-1 (Ruixinbio, RXM2D202486, China), mouse TGF-α (Ruixinbio, RX202404M, China), human IL-1β (Ruixinbio, RX106152H, China), human IL-18 (Ruixinbio, RX106154H, China), human IL-6 (Ruixinbio, RX106126H, China), human MCP-1 (Ruixinbio, RXG67833, China), and human TGF-α (Ruixinbio, RX104770H, China) were measured strictly according to the instructions of the respective ELISA kits.

### Oil red O staining

Aortas were collected from the base of the ascending aorta to the iliac bifurcation for measurement of aortic en face atherosclerotic lesions for quantification of the atherosclerotic lesion area. Whole aortas were stained with 0.5% oil red O (Solarbio, G1260, China) for 30 min to detect the aortic lesion burden. The positively stained lesion areas were quantified via ImageJ, and the data are presented as the percentage of oil red O-positive lesion areas relative to the total aortic area.

### Hematoxylin and eosin (HE) staining

The aortic arch segments between the first and second branches were processed for paraffin embedding and sectioned at a thickness of 4 μm, with serial sections systematically numbered for histological analysis. For each experimental group, matched serial sections were selected for parallel staining with HE to evaluate the plaque area. Briefly, the sections were stained with hematoxylin (Solarbio, G1080, China) for 15 min after routine deparaffinization and dehydration. Prior to eosin (Solarbio, G1100, China) staining, the sections were treated with 1% hydrochloric acid alcohol (Biosharp, BL638A, China). Images were collected via NiE (Nikon, Japan) after dehydration, clearing, and sealing. The total plaque area (μm²) was measured by thresholding eosinophilic regions, excluding the media/adventitia, via ImageJ.

### Tissue immunofluorescence (IF)

For IF, frozen sections of mouse aorta tissue were rewarmed for 30 min. Then, the sections were washed with PBS three times, subjected to heat-mediated antigen retrieval via Tris-EDTA buffer (Beyotime, R0225, China), blocked (30 min, 37 °C) with 5% BSA (Biofoxx, 4240GR500, Germany), and incubated with primary antibodies overnight at 4 °C. The primary antibodies used were as follows: anti-NLRP3 rabbit antibody (Proteintech, 19771-1-AP, China, 1:100), anti-TRPV4 (ABclonal, A5660, China, 1:100), anti-CD68 mouse antibody (Servicebio, GB14043, China, 1:200), anti-α-SMA mouse antibody (Servicebio, GB13044, China, 1:200), anti-CD31 goat antibody (R&D, AF3628, USA, 1:200), anti-TOMM20 (Servicebio, GB111481, China, 1:200), and anti-LC3B (Servicebio, GB113801, China, 1:200). Finally, the tissue sections were incubated with the following species-specific secondary antibodies (60 min, 37 °C, dark): goat anti-rabbit IgG-Alexa Fluor 594 (Invitrogen, A-11012, USA, 1:500), goat anti-mouse IgG-Alexa Fluor 647 (Jackson, 115-605-003, USA, 1:500), and donkey anti-goat IgG-Alexa Fluor 647 (Invitrogen, A-21447, USA, 1:500), followed by nuclear counterstaining (10 min, 37 °C, dark) with 4’,6-diamidino-2-phenylindole (DAPI) (Solarbio, C0060, China, 1:100).

### Vascular permeability assays

Vascular permeability was evaluated via the use of evans blue (MAokangbio, MS4007, China) as previously described.^47^ After PEMF intervention, sterile 0.5% evans blue solution was injected into the caudal vein of each group. Thirty minutes later, the mice were euthanized, and the aorta was harvested. Researchers took representative images of blue exudation differences and measured the tissue weight. The concentration of evans blue in the aortas of different groups of mice was quantified by measuring the absorbance at 610 nm.

### Untargeted lipidomics

Aortic tissues from three experimental groups (*n* = 5 mice in each group) were homogenized in ice-cold butanol:methanol (1:1, v/v) for lipid extraction. Lipid profiling was performed via a SHIMADZU Nexera LC-30 UHPLC system coupled with a Q Exactive Plus mass spectrometer (Thermo, USA) in both positive and negative ionization modes. Chromatographic separation was achieved on a Hypersil GOLD™ C18 column (Thermo, 25003-102130, USA) with a 17-min gradient (mobile phase A: 10 mM ammonium formate in acetonitrile: water (4:6, v/v); B: acetonitrile: isopropanol (1:9, v/v)). The raw data were processed via MS-DIAL software (v4.7) for peak detection, alignment, and lipid identification against an in-house database. Untargeted lipidomics and analysis were performed by Shanghai Bioprofile Technology.

### Cell death assay

Pyroptotic cell death was evaluated with an LDH release assay and Hoechst 33342/PI staining. For LDH release, HUVECs (1 × 10^4^ cells/well) were plated in 96-well plates and allowed to adhere overnight. Following Ox-LDL or PEMF treatment, the supernatant from each well was harvested and divided into three aliquots. LDH release was detected via an LDH assay kit (Beyotime, C0017, China). Briefly, 25 μL of cell supernatant and 25 μL of substrate were mixed together and added to the mixture (15 min, 37 °C, dark). Then, 25 μL of 2,4-dinitrophenylhydrazine was added to the samples (15 min, 37 °C, dark). Finally, 250 μL of 0.4 mol/L NaOH solution was added (5 min, 37 °C, dark). The absorbance was measured at 450 nm on a spectrophotometric microplate reader (Bio-Tek, SYNERGY H1, USA). For Hoechst 33342/PI staining (Beyotime, C1056, China), HUVECs, HAECs or MAECs (1 × 10^5^ cells/well) were plated in a 12-well plate and treated with Ox-LDL or PEMFs for the duration specified in the appropriate section. The cell suspensions were collected and incubated with a mixed solution of Hoechst 33342 and PI (25 min, 37 °C, dark). The fluorescence signals were then analyzed via flow cytometry (Beckman Coulter, CytoFLEX, USA), with blue fluorescence and red fluorescence detected through appropriate optical filters.

### CCK8 assay

HUVECs (1 × 10^4^ cells/well) were plated in 96-well plates according to the experimental groups and requirements, with a total of three wells per group. After the modeling and intervention were completed, the original culture medium in the wells was discarded, warmed PBS was added to gently wash the cells, and the CCK8 (Beyotime, C0038, China) working solution prepared in advance (CCK8: F12K serum-free medium = 1:10) was added (60 min, 37 °C, dark) to each well. The absorbance value at 450 nm was measured with a microplate reader (Bio-Tek, SYNERGY H1, USA).

### JC-1 assay

Experiments were performed via an enhanced mitochondrial membrane potential detection kit (Beyotime, C2003S, China). HUVECs (1 × 10^4^ cells/well) were plated in high-throughput dedicated 96-well plates. After the disease model and intervention treatment were completed, the original medium was discarded, and preheated PBS solution was added for gentle cleaning once. One hundred microlitres of fresh medium and 100 μL of JC-1 staining working solution were added to each well, mixed well, and incubated (20 min, 37 °C, dark). After the incubation was complete, the staining solution was discarded, JC-1 staining buffer was added, and the samples were gently washed twice. Finally, 100 μL of PBS solution was added for high-throughput on-machine testing. After testing, the built-in analysis software of the high-throughput multiparameter cell dynamic analysis system was used to analyze the intensity of red and green fluorescence (PerkinElmer, USA).

### Detection of reactive oxygen species (ROS)

A ROS assay kit (Beyotime, S0035S, China) was used to detect the accumulation of ROS in ECs according to the manufacturer’s instructions. Briefly, HUVECs (1 × 10^4^ cells/well) were plated in 96-well plates and allowed to adhere overnight. Following Ox-LDL or PEMF treatment, HUVECs were loaded with DCFH-DA (10 μM, 20 min, 37 °C, dark) in serum-free medium, followed by three washing steps with PBS. The fluorescence intensity was detected with a fluorescence microplate reader (Leica, Germany).

### Detection of mitochondrial reactive oxygen species (mtROS)

The accumulation of mtROS in ECs was detected via a mitochondrial superoxide fluorescent probe (Maokangbio, MX4313, China). Briefly, HUVECs (1 × 10^4^ cells/well) were plated in 96-well plates dedicated for high-throughput analysis and allowed to adhere overnight. Following Ox-LDL or PEMF treatment, HUVECs were loaded with staining working solution (5 μM, 10 min, 37 °C, dark) in serum-free medium, and then Hoechst (Beyotime, C1027, China) staining solution was added (10 min, 37 °C, dark), followed by three washing steps with PBS for high-throughput on-machine testing. After testing, the fluorescence intensity was analyzed via the built-in analysis software of the high-throughput multiparameter cell dynamics analysis system (PerkinElmer, USA).

### Detection of intracellular calcium levels

A Fluo-4 AM calcium fluorescent probe (Beyotime, S1060, China) was used to detect intracellular calcium levels. Briefly, HUVECs (1 × 10^4^ cells/well) were plated in 96-well plates and loaded with Fluo-4 AM working solution (1 μM, 30 min, 37 °C, dark) in serum-free medium, followed by three washing steps with PBS. The fluorescence intensity was detected with a fluorescence microplate reader (Leica, Germany).

### Detection of mitochondrial autophagy

Mitochondrial autophagy was detected via a detection kit (DOJINDO, MD01, Japan). In brief, HUVECs (1 × 10^5^ cells/well) were plated in 12-well plates. Following Ox-LDL or PEMF treatment, HUVECs were loaded with 100 nmol/L Mtphagy Dye staining working solution (30 min, 37 °C, dark). The fluorescence intensity of the arthropod dye was measured via flow cytometry (Beckman Coulter, CytoFLEX, USA).

### Detection of membrane tension

Membrane tension was assessed via a Flipper-TR® fluorescent cell membrane tension probe (Cytoskeleton, CY-SC020, USA). Briefly, HUVECs (1 × 10^5^ cells/well) were plated in 15-mm confocal dishes and loaded with staining working solution (1 μM, 15 min, 37 °C, dark) in serum-free medium, followed by three washing steps with PBS. After incubation was completed, the confocal dishes were removed and subjected to on-machine testing via a STED ultrahigh-resolution microscope (Leica, STELLARIS 5 STED, Germany). The testing method employed standard fluorescence lifetime imaging microscopy technology.

### Atomic Force Microscopy (AFM)

#### In vitro

The cell morphology (height, length, width) and elastic modulus were assessed via Dimension icon AFM (Bruker Corporation, Germany). Briefly, HUVECs (8 × 10^5^ cells/well) were seeded in 6 cm cell culture dishes. Silicon probes (Bruker Corporation, RTESP-150, Germany; spring constant: 5 N/m; tip radius: 8 nm) with a spherical borosilicate glass probe 5 µm in diameter were employed to obtain AFM topography images and elastic moduli. Images (512 × 512 pixels) were acquired at a 2 μm/s scan rate. The Young’s modulus was calculated by fitting the retraction curves via the Hertz contact model via NanoScope Analysis 3.0 software. A minimum of 10 cells per experimental group were analyzed, with at least 10 force curves collected per cell.

#### In vivo

Aortic segments (6 mm in length) were harvested from the mice in each group, longitudinally incised, flattened and fixed on glass slides via a biocompatible adhesive (Cell-Tak, 354240, Corning, USA), with the intimal surface facing upward. In situ detection of viable aortic ECs was performed via AFM (NanoWizard 4 XP, Bruker Corporation, Germany). A silicon probe (Bruker Corporation, SNL10, Germany; spring constant: 0.35 N/m; tip radius: 2 nm) equipped with a spherical tip (diameter: 10 μm) was used. The automated probe approach was conducted at a speed of 2 μm/s, and the samples were imaged in contact mode at room temperature. The scanning range was 50 μm × 50 μm, with data acquisition points arranged in a 3 × 3 matrix at intervals of 1 μm × 1 μm, and approximately 30 force curves were collected per point. The force curve results were fitted via the Hertz model to calculate the Young’s modulus.

### Electrophysiology

For whole-cell patch-clamp recordings, HUVECs were plated on 5 mm round glass coverslips coated with poly-D-lysine and type I collagen and maintained in a humidified atmosphere at 37 °C for 24 h. All patch-clamp recordings were performed at room temperature (~24 °C). Whole-cell currents were recorded via a Multiclamp 700B amplifier (Molecular Devices, USA) coupled with a Digidata 1440 A data acquisition system (Molecular Devices, USA). Patch pipettes were pulled from borosilicate glass capillaries (Sutter Instrument, BF150-86-10, USA) via a P-97 micropipette puller (Sutter Instrument, USA), with a tip resistance of 5–7 MΩ when filled with intracellular solution. The bath solution contained (in mM) 140 NaCl, 5 KCl, 2 CaCl₂, 1 MgCl₂, 10 glucose, and 10 HEPES (pH adjusted to 7.4 with 1 M NaOH). The pipette mixture consisted of (in mM) 140 KCl, 5 EGTA, 2 Mg-ATP, 0.1 Na_2_-GTP, and 10 HEPES (pH adjusted to 7.2 with 1 M KOH). After establishing the whole-cell configuration, the cells were allowed to stabilize for 5 min before data recording. TRPV4-mediated currents were elicited by a voltage ramp protocol from −100 mV to +100 mV applied every 10 s from a holding potential of −70 mV. The data were sampled at 10 kHz and filtered at 3 kHz via pCLAMP 10.7 software (Molecular Devices, USA). For pharmacological interventions, the TRPV4 agonist GSK1016790A (80 nM) or the antagonist HC067047 (100 nM) was applied via a gravity-driven perfusion system (flow rate: 1 mL/min). The data were analyzed via Clampfit 11.2 (Molecular Devices, USA) and GraphPad Prism 9. Whole-cell patch-clamp recordings and analysis were performed by BioMed Scientific (Wuhan, China).

### Transmission electron microscopy (TEM)

The trimmed aortic arch samples or HUVECs were sequentially fixed with 3% glutaraldehyde (primary fixation) and 1% osmium tetroxide (postfixation). Tissue dehydration was performed using graded acetone concentrations (30%, 50%, 70%, 80%, 90%, 95%, and 100%). Following dehydration, the samples were infiltrated with an epoxy resin mixture (dehydrant:embedding agent = 1:1, then pure embedding agent) and polymerized. Ultrathin sections (70–90 nm) were prepared via an ultramicrotome, collected on copper grids, and stained with uranyl acetate (10–15 min, 37 °C) followed by lead citrate (1–2 min). TEM imaging was conducted using a Hitachi HT-7800 system operated at 80 kV. TEM imaging was performed by Chengdu Lilai Biotechnology Co., Ltd.

### Detection of mitochondrial permeability transition pore (mPTP)

The mPTP opening was assessed via a commercial kit (Beyotime, C0265). Briefly, HUVECs (1 × 10^5^ cells/well) were plated in 12-well plates. Following Ox-LDL or PEMF treatment, HUVECs were loaded with 0.5× calcein AM staining solution and 1× fluorescence quenching solution (30 min, 37 °C, dark) and analyzed via flow cytometry (CytoFLEX, FL1 channel).

### Detection of the oxygen consumption rate (OCR)

The OCR was assessed via a Seahorse XF cell mito stress test kit (Agilent, 103015-100, USA). Briefly, HUVECs or MAECs (1.5 × 10^5^ cells/well) were plated in Seahorse XF Pro M cell culture microplates (Agilent, 103774-100, USA). Following Ox-LDL or PEMF treatment, the medium was replaced with XF assay medium, followed by incubation at 37 °C for 1 h in a CO_2_-free incubator. The basal OCR and the OCR following the addition of oligomycin (1 µM), FCCP (0.5 µM), and rotenone/antimycin A (0.5 µM) were measured via a Seahorse XF Pro analyzer (Agilent, S7859AN, USA). Protein concentrations were determined with a BCA protein assay kit, and the OCR was normalized to the total protein concentration. The OCR was analyzed via Wave Pro software.

### F-actin staining

HUVECs (2 × 10^5^ cells/well) were plated in 24-well cell culture plates. The cells were washed with PBS, fixed with 4% paraformaldehyde, and incubated with rhodamine phalloidin (ABclonal, RM02835, China; 1:100) for 1 h. The cell nuclei were stained with DAPI.

### Western blot

The animals were sacrificed 3 weeks after PEMF treatment, and aortic tissue was harvested. HUVECs or HAECs (8 × 10^5^ cells/well) were seeded in 6 cm cell culture dishes, and the cells were collected after Ox-LDL or PEMF treatment. Homogenates from aorta tissue, HUVECs or HAECs were lysed in radioimmunoprecipitation assay buffer (Beyotime, P0013B, China) containing protease (MCE, HY-K0010, USA) and phosphatase inhibitor cocktails (MCE, HY-K0021, HY-K0022, USA) at 4 °C. Protein concentrations were determined via a bicinchoninic acid (BCA) kit (Beyotime, P0009, China). After normalization, the samples were subjected to 10% SDS‒PAGE and then transferred to a PVDF membrane (Millipore, USA). These membranes were blocked with 5% nonfat dry milk (Yili Milk Company, China) for 1 h at room temperature and incubated with primary antibodies against TRPV4 (ABclonal, A5660, China, 1:1000), NLRP3 (Proteintech, 19771-1-AP, China, 1:1000), ASC (CST, 67824, USA, 1:1000), p20 caspase-1 (AdipoGen, AG-20B-0042, USA, 1:1000), GSDMD (HUABIO, ER1901-37, China, 1:1000), AIM2 (ABclonal, A22874, China, 1:1000), NLRC4 (ABclonal, A7382, China, 1:1000), TRPC1 (ABclonal, A12525, China, 1:1000), ICAM-1 (Proteintech, 10831-1-AP, China, 1:1000), and β-actin (Abways, AY0573, China, 1:10,000) at 4 °C overnight. After being washed with Tris-buffered saline plus Tween 20 (TBST), the membranes were incubated with anti-rabbit immunoglobulin G (IgG) horseradish peroxidase-conjugated secondary antibodies (ZSGB-Bio, ZB-2301, China, 1:25,000) for 1 h. Immunoreactive bands were visualized via an enhanced chemiluminescent detection system (Bio-Rad, USA). The immunoreactive bands were quantified by densitometric analysis via ImageJ software.

### Statistical analysis

The results are expressed as the mean ± SEM. Statistical tests were performed with GraphPad Prism 9. The Kolmogorov‒Smirnov test, the Shapiro‒Wilk normality test, and the normal probability (Q‒Q) plot were used to determine the normality of the data. For normally distributed variables, the F test was used to test for homogeneity of variance for 2-group comparisons, the unpaired Student’s *t* test was used for data with homogenous variance, and the unpaired t test with Welch correction was utilized for data with heterogeneous variance. The Bartlett and Brown-Forsythe tests were used to test for homogeneity of variance for multigroup comparisons. One-way ANOVA with Tukey’s multiple comparison test was used for data with homogenous variance, and Brown–Forsythe and Welch ANOVA tests followed by Dunnett’s multiple comparison test were used for heterogeneous data. *p* values less than 0.05 indicated statistical significance. All tests were performed at least 3 times.

## Supplementary information

Supplementary materials

## Data Availability

All data supporting the findings of this study are available within the article and its supplementary materials.
