# Supplementary material for: Pulsed electromagnetic fields inhibit atherosclerosis by regulating pyroptosis through membrane tension-mediated mechanosensitive channels
Source: Signal Transduct Target Ther. 2025 Nov 28;10:388. doi: 10.1038/s41392-025-02479-2 (PMC12660931; doi:10.1038/s41392-025-02479-2)
Supplement: Supplementary file 1 — Supplementary materials [file 41392_2025_2479_MOESM1_ESM.docx]

Supplementary Materials for

Pulsed electromagnetic fields inhibit atherosclerosis by regulating pyroptosis through membrane tension-mediated mechanosensitive channels

Hongxin Cheng^1, 2#^, Qing Zhang^1, 2#^, Wen Zhong^1, 2^, Hanbin Li^1, 2^, Lu Wang^1, 2^, Shiqi Wang^1, 2^, Chengqi He^1, 2^, Chenying Fu^3, 4*^, Quan Wei^1, 2*^

Correspondence to: Dr. Quan Wei, E-mail: [weiquan@scu.edu.cn](mailto:weiquan@scu.edu.cn) and Dr. Chenying Fu, E-mail: [fcying_2004@163.com](mailto:fcying_2004@163.com)

**This PDF file includes:**

Figure. S1 to S10

Table. S1

**
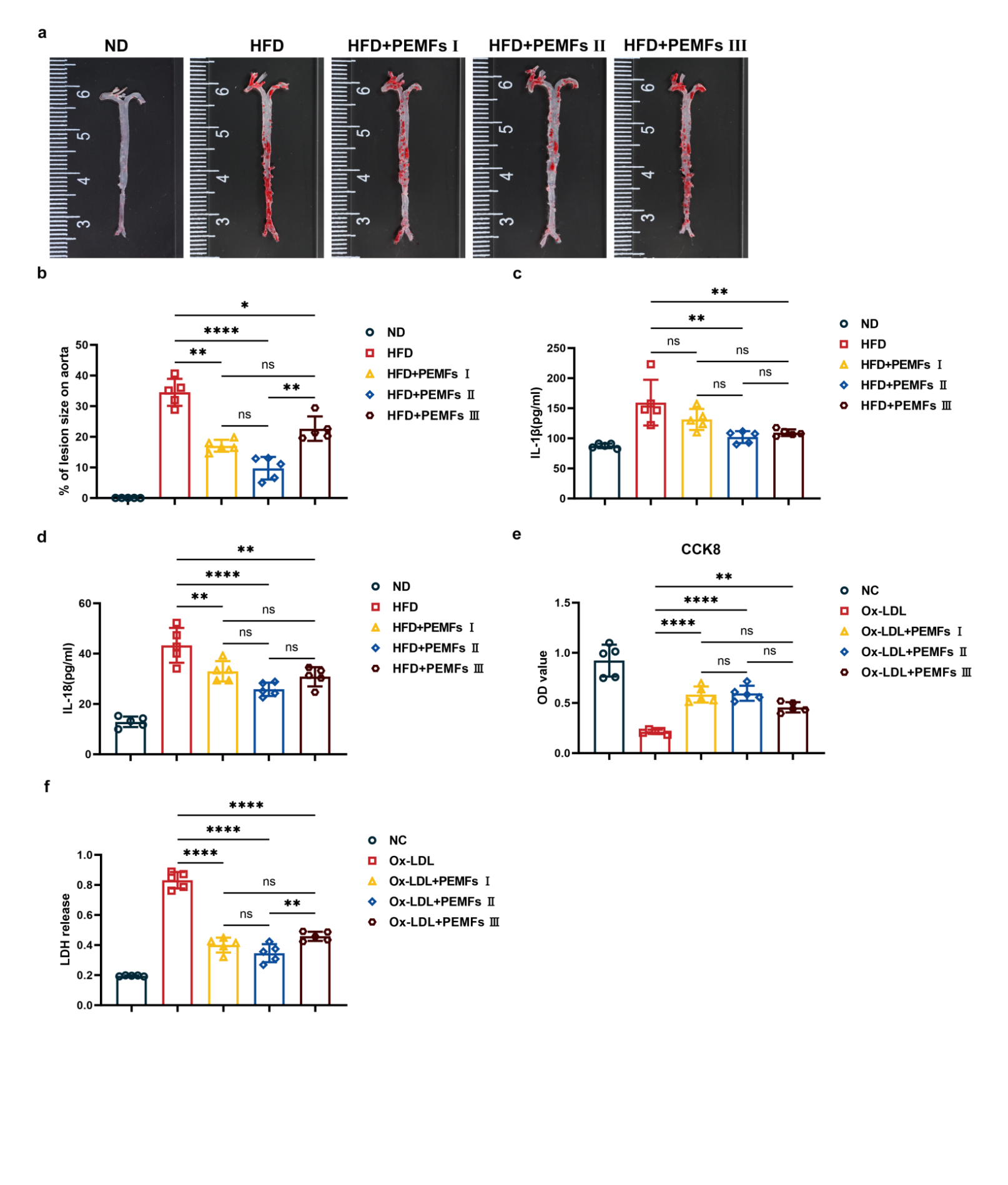
**

**Supplementary Figure 1. 15 Hz, 1.5 mT PEMFs exhibit superior efficacy in suppressing atherosclerotic plaque formation.**

a, Oil red O staining was used to show the lesion burden in the aorta in ApoE-/- mouse groups: normal chow diet (ND) group, high-fat diet (HFD,15 weeks) group, HFD+PEMFs I (5 Hz, 0.5 mT, 1 h/day, 3 weeks) group, HFD+PEMFs II (15 Hz, 1.5 mT, 1 h/day, 3w) group, n=5 mice per group. b, Quantitative analysis of representative images of oil red O staining, n=5 mice per group. c and d, Serum levels of IL-1β and IL-18 were detected by ELISA, n=5 mice per group. e, CCK8 was used to detect cell viability in HUVEC groups: normal cell (NC) group, Ox-LDL (100 μg/mL, 24 h) group, Ox-LDL+PEMFs I (5 Hz, 0.5 mT, 1 h/day, 1d) group, Ox-LDL+PEMFs II (15 Hz, 1.5 mT, 1 h/day, 1d) group, and Ox-LDL+PEMFs III (30 Hz, 3 mT, 1 h/day, 1d) group, n=5 independent experiments per group. f, LDH release level in the supernatant of HUVECs was detected by the LDH kit, n=5 independent experiments per group. All data represent biological replicates. The measured data were presented as the mean ± SEM. Statistical significance was assessed by one-way ANOVA with Tukey’s multiple comparison test. **p*<0.05，***p*<0.01，****p*<0.001，*****p*<0.0001.

**
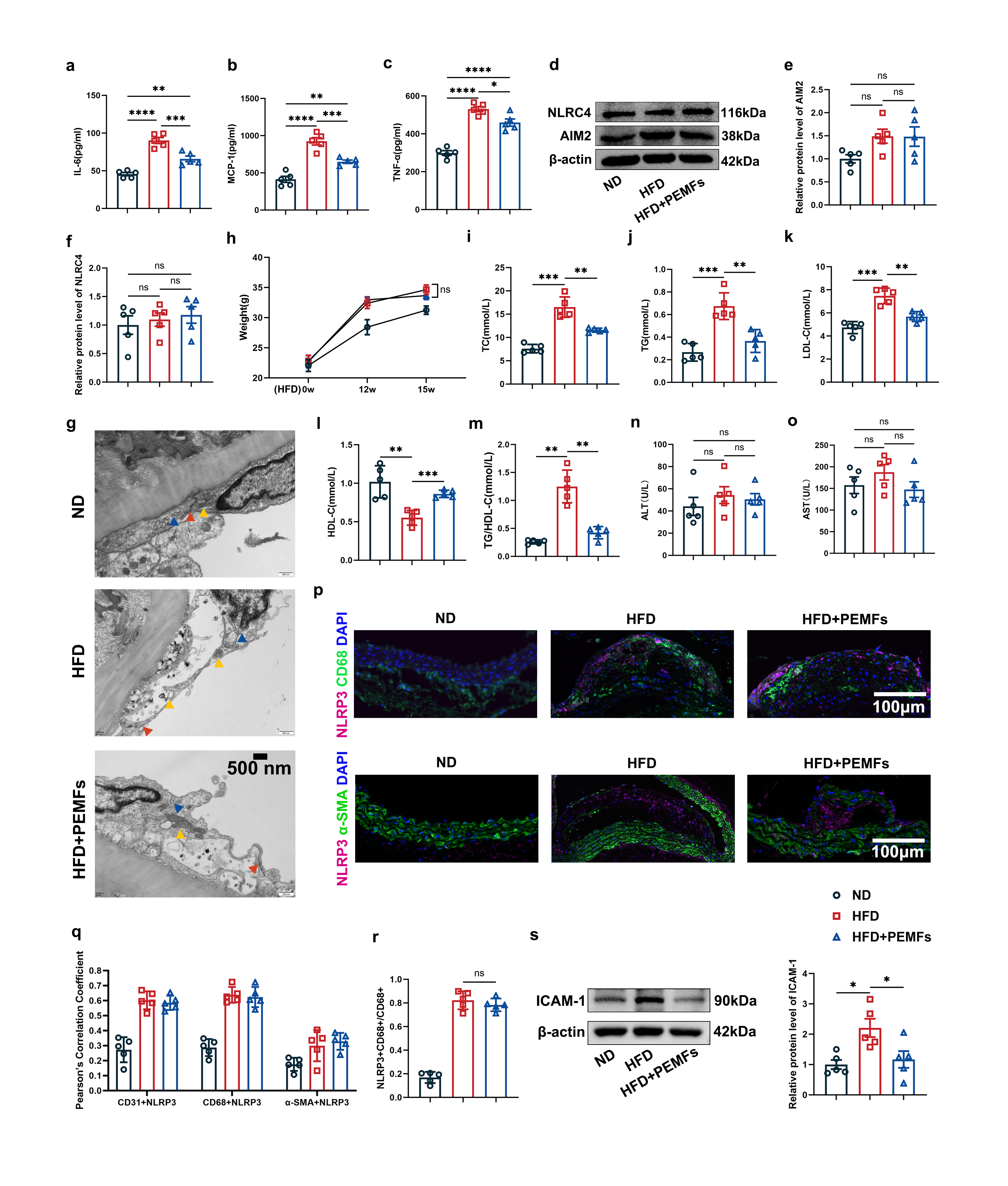
**

**Supplementary Figure 2. PEMFs inhibit the formation of atherosclerotic plaques via attenuating pyroptosis and inflammation.**

a through c, Serum levels of IL-6, MCP-1, and TNF-α were detected by ELISA in ApoE-/- mouse groups: normal chow diet (ND) group, high-fat diet (HFD, 15 weeks) group, and HFD+PEMFs (15 Hz, 1.5 mT, 1 h/day, 3 weeks) group, n=5 mice per group. d through f, Western blot analysis of NLRC4 and AIM2 protein expression in aortic tissues, n=5 mice per group. g, Transmission electron microscopy images of vascular endothelial cells in the mouse aortic arch. Yellow arrows: mitochondria; blue arrows: endoplasmic reticulum; red arrows: intercellular space. n=5 mice per group. Scale bar=500 nm. h, Body weights of ApoE-/- mice were recorded at the start of HFD (0 weeks), after 12 weeks of HFD, and after 15 weeks (following PEMFs intervention), n=20 mice per group. i through m, Serum levels of TC, TG, LDL-C, HDL-C, and TG/HDL-C was detected by a biochemical analyzer, n=5 mice per group. n and o, Serum levels of ALT and AST was detected by a biochemical analyzer, n=5 mice per group. p, Double immunostaining of NLRP3 with CD68 and α-SMA in mouse atherosclerotic lesions, n=5 mice per group. Scale bar=100 μm. q, Pearson correlation coefficient (PCC) analysis of NLRP3 colocalization with CD31, CD68, and α-SMA. A PCC value>0.5 indicates significant colocalization, n=5 mice per group. r, Manders' coefficient quantifying the proportion of NLRP3-positive areas within CD68-positive regions in the aortic arch plaques, n=5 mice per group. s, Western blot analysis and quantitative analysis of ICAM-1 protein expression in aortic tissues, n=5 mice per group. All data represent biological replicates. The measured data were presented as the mean ± SEM. Statistical significance was assessed by one-way ANOVA with Tukey’s multiple comparison test. **p*<0.05，***p*<0.01，****p*<0.001，*****p*<0.0001.


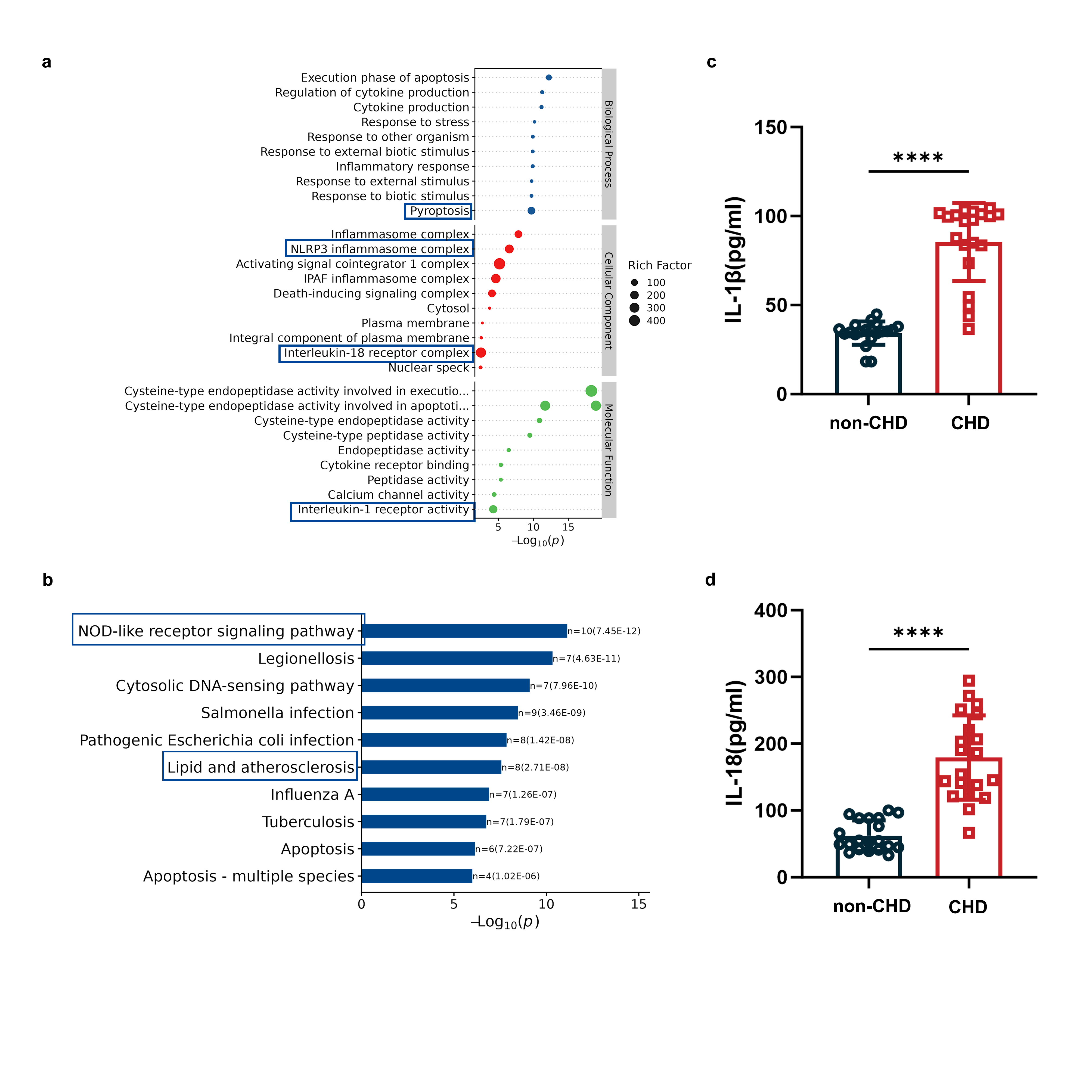


**Supplementary Figure 3.** **NLRP3 inflammasome mediated pyroptosis is indeed a key factor in the progression of atherosclerosis.**

a, GO analysis of differentially expressed proteins between non-coronary heart disease (CHD) group and CHD group, showing enrichment in biological processes (blue), cellular components (red), and molecular functions (green), n=20 persons in each group. b, KEGG pathway enrichment analysis of differentially expressed proteins, n=20 persons per group. c and d, The levels of IL-1β and IL-18 in the human plasma were detected by ELISA, n=20 persons per group. The measured data were presented as the mean ± SEM. Statistical significance was assessed by Student t test. **p*<0.05，***p*<0.01，****p*<0.001，*****p*<0.0001.


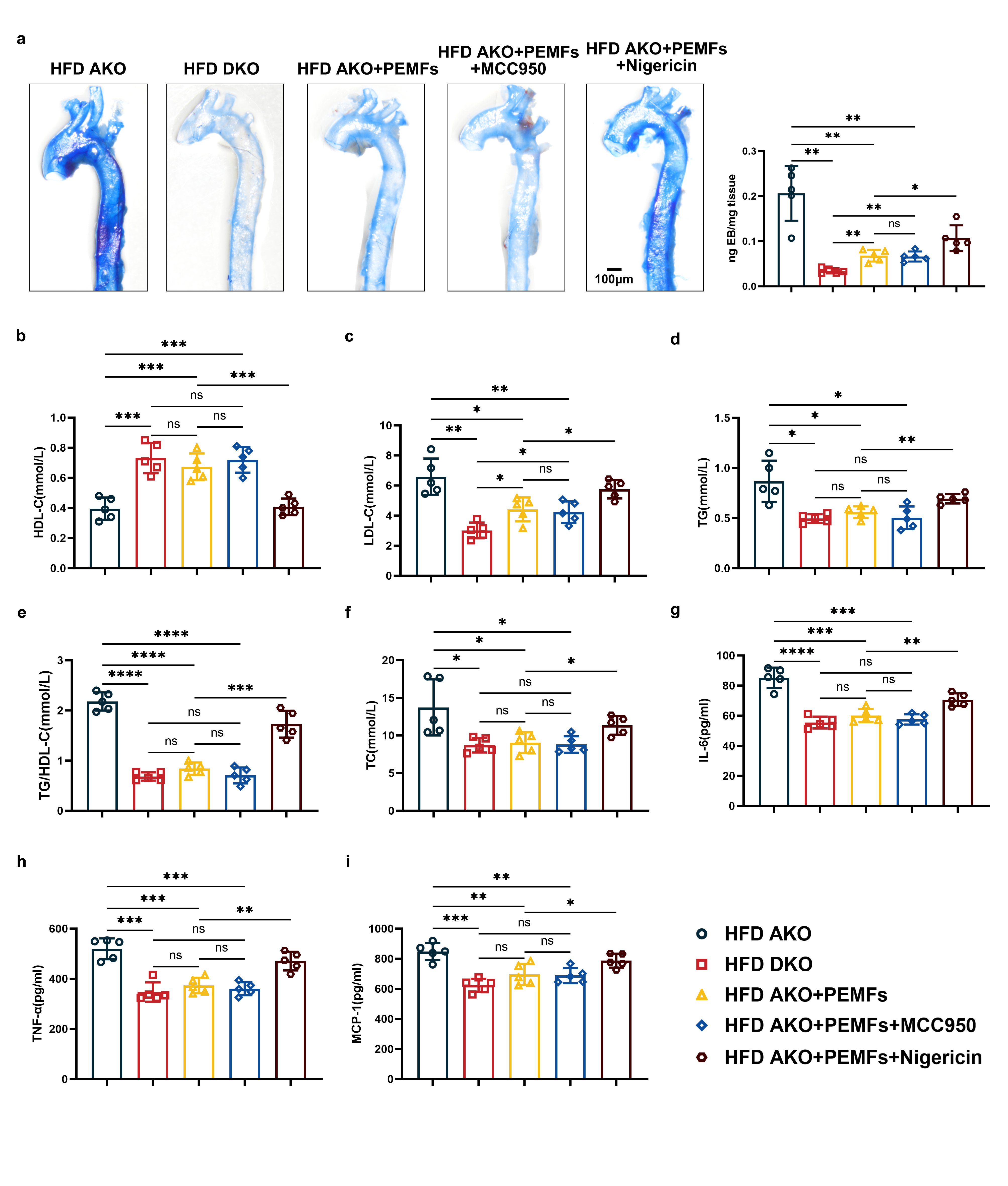


**Supplementary Figure 4. NLRP3 suppression alleviated inflammation and vascular injury, contrasting with NLRP3 overactivation which aggravated these pathological processes.**

a, Evans blue staining was used to reveal aortic permeability in ApoE-/- (AKO) mouse and ApoE-/-NLRP3-/- (DKO) mouse groups: HFD AKO group, HFD DKO group, HFD AKO+ PEMFs group, HFD AKO+PEMFs+MCC950 group, and HFD AKO+PEMFs+Nigericin group, n=5 mice per group. Scale bar=100 μm. b through f, Serum level of HDL-C, LDL-C, TG, TG/HDL-C, and TC was detected by a biochemical analyzer, n=5 mice per group. g through i, Serum levels of IL-6, TNF-α, and MCP-1 were detected by ELISA, n=5 mice per group. All data represent biological replicates. The measured data were presented as the mean ± SEM. Statistical significance was assessed by one-way ANOVA with Tukey’s multiple comparison test. **p*<0.05，***p*<0.01，****p*<0.001，*****p*<0.0001.


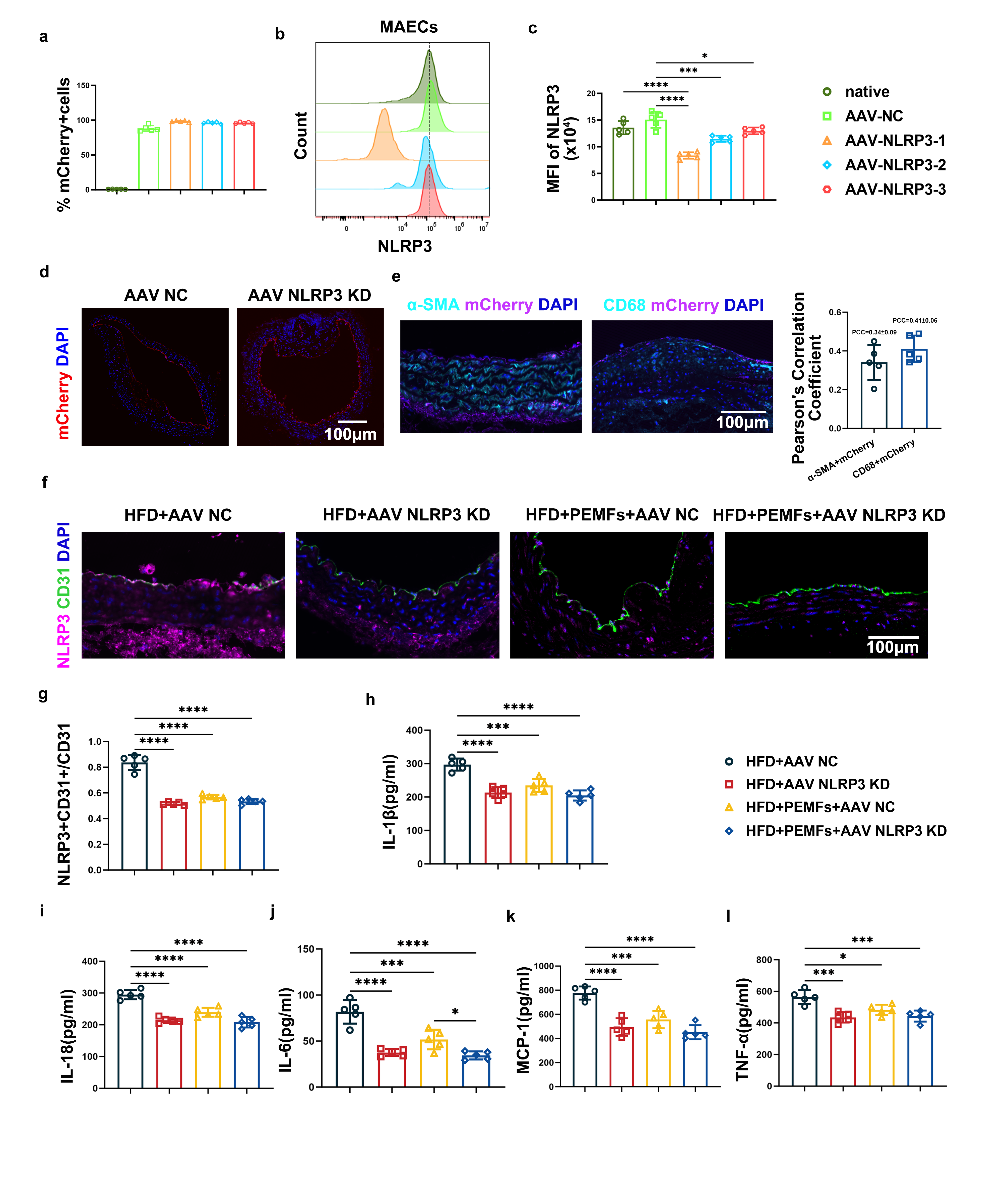


**Supplementary Figure 5. Endothelial-specific NLRP3 knockdown attenuates inflammatio.**

a, mCherry positive cell rate was detected by flow cytometry in MVECs isolated from heart and aortic tissues of each group: native group, AAV NC group, AAV NLRP3-1 group, AAV NLRP3-2 group, and AAV NLRP3-3 group, n=5 mice per group. b and c, MFI of NLRP3 was measured by flow cytometry, n=5 mice per group. d, mCherry was observed by immunofluorescence analysis, n=5 mice per group. e, Pearson correlation coefficient (PCC) analysis of mCherry colocalization with CD68 and α-SMA. A PCC value>0.5 indicates significant colocalization, n=5 mice per group. f and g, Manders' coefficient quantifying the proportion of NLRP3-positive areas within CD31-positive regions in the aortic arch plaques in each group: HFD+AAV NC group, HFD+AAV NLRP3 KD group, HFD+PEMFs+AAV NC group, and HFD+PEMFs+NLRP3 KD group, n=5 mice per group. h through l, Serum levels of IL-1β, IL-18, IL-6, MCP-1, and TNF-α were detected by ELISA, n=5 mice per group. All data represent biological replicates. The measured data were presented as the mean ± SEM. Statistical significance was assessed by one-way ANOVA with Tukey’s multiple comparison test. **p*<0.05，***p*<0.01，****p*<0.001，*****p*<0.0001.

**
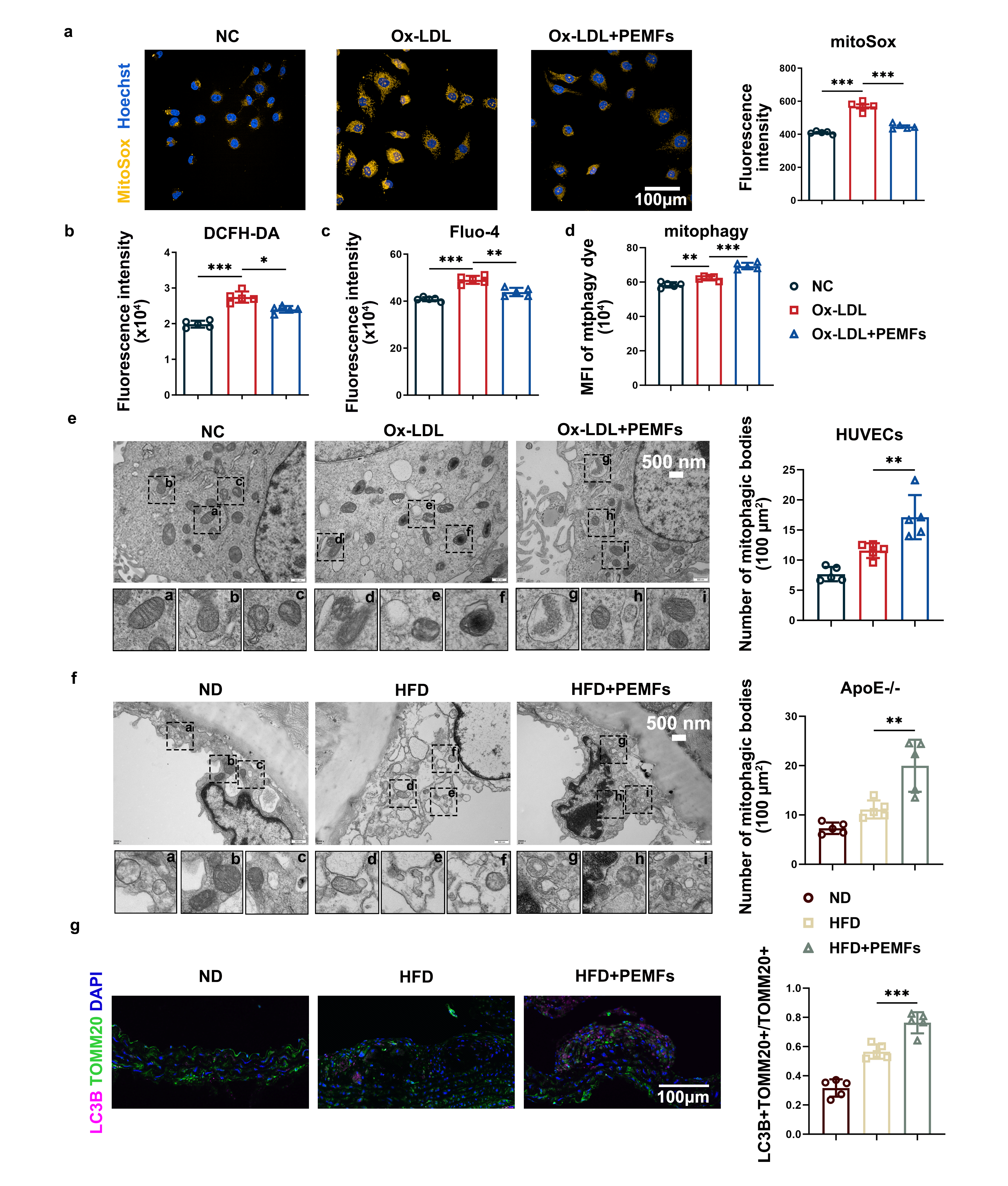
**

**Supplementary Figure 6. PEMFs regulate mitochondrial dysfunction.**

a, Mitochondrial reactive oxygen species (ROS) levels was detected by mitoSox staining in HUVEC groups: normal cell (NC) group, Ox-LDL (100 μg/mL, 24 h) group, and Ox-LDL+ PEMFs (15 Hz, 1.5 mT, 1 h/day, 1d) group, n=5 independent experiments per group. Scale bar=100 μm. b, Cellular superoxide anion levels were detected by DCFH-DA, n=5 independent experiments per group. c, Intracellular calcium ion levels were detected using Fluo-4 AM in HUVECs, n=5 independent experiments per group. d, Mitophagy assessed by flow cytometry using a mitophagy detection kit in HUVECs, n=5 independent experiments per group. e, Mitochondrial morphology and mitophagy were observed by TEM in HUVECs, n=5 independent experiments per group. f, Mitochondrial morphology and mitophagy were observed by TEM in ApoE-/- mice, n=5 mice per group. g, Double immunostaining of LC3B with TOMM20 in mouse atherosclerotic lesions, and Manders' coefficient analysis of LC3B colocalization with TOMM20, n=5 mice per group. Scale bar=100 μm. All data represent biological replicates. The measured data were presented as the mean ± SEM. Statistical significance was assessed by one-way ANOVA with Tukey’s multiple comparison test. **p*<0.05，***p*<0.01，****p*<0.001，*****p*<0.0001.


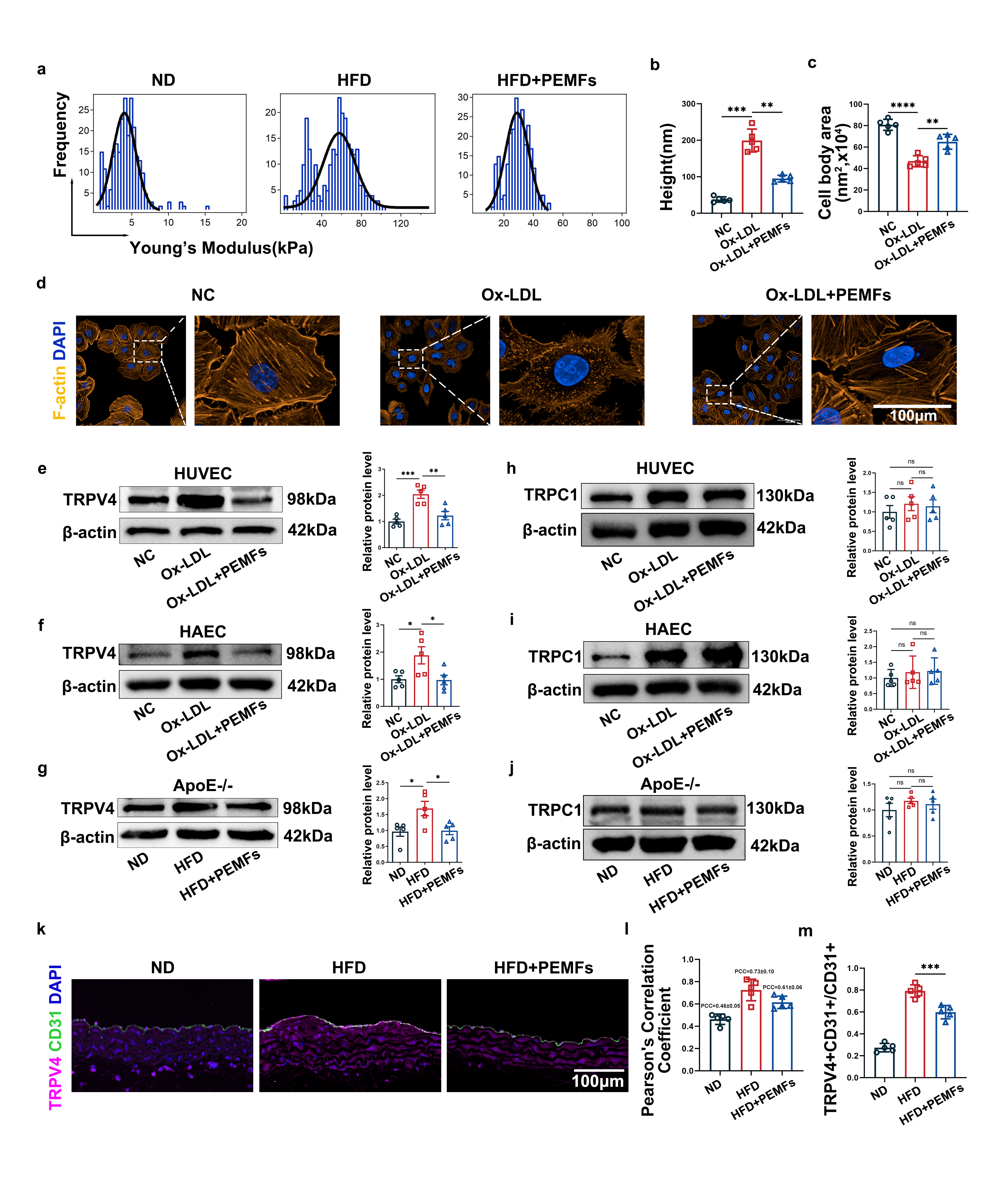


**Supplementary Figure 7. ECs membrane tension and TRPV4 participated in endothelial injury.**

a, Histograms of the slope obtained from the load curves of force volume images in the ND, HFD and HFD+PEMFs groups. The slopes indirectly reflect the stiffness of the aortic intima in AFM, n=5 mice per group. b and c, Cell height and cell body area were calculated from topographical images of HUVECs, n=5 independent experiments per group. d, Cytoskeleton morphology was observed by F-actin (orange) and DAPI (blue) staining, n=5 independent experiments per group. Scale bar=100 μm. e, Western blot analysis of TRPV4 protein expression in HUVECs, n=5 independent experiments per group. f, Western blot analysis of TRPV4 protein expression in HAECs, n=5 independent experiments per group. g, Western blot analysis of TRPV4 protein expression in aortic tissues, n=5 mice per group. h, Western blot analysis of TRPC1 protein expression in HUVECs, n=5 independent experiments per group. i, Western blot analysis of TRPC1 protein expression in HAECs, n=5 independent experiments per group. j, Western blot analysis of TRPC1 protein expression in aortic tissues, n=5 mice per group. k, Double immunostaining of TRPV4 with CD31 in mouse atherosclerotic lesions, Scale bar=100 μm. l, PCC analysis of TRPV4 colocalization with CD31, n=5 mice per group. m, Manders' coefficient quantifying the proportion of TRPV4-positive areas within CD31-positive regions in the aortic arch plaques, n=5 mice per group. All data represent biological replicates. The measured data were presented as the mean ± SEM. Statistical significance was assessed by one-way ANOVA with Tukey’s multiple comparison test. **p*<0.05，***p*<0.01，****p*<0.001，*****p*<0.0001.

**
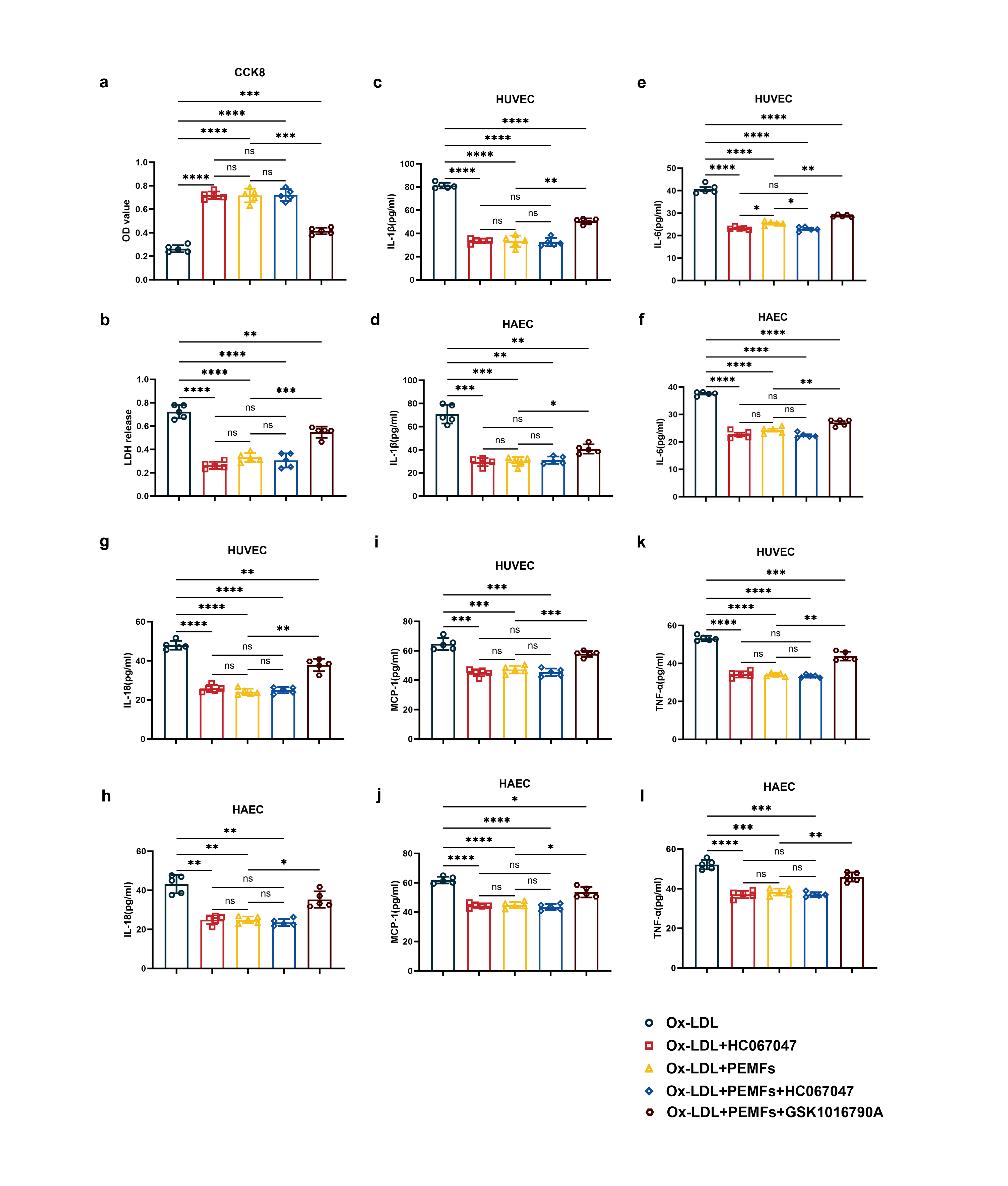
**

**Supplementary Figure 8. TRPV4 suppression alleviated inflammation, contrasting with TRPV4 overactivation which aggravated these pathological processes in HUVECs or HAECs.**

a, CCK8 was used to detect cell viability in HUVEC groups: Ox-LDL group, Ox-LDL+ HC067047 group, Ox-LDL+PEMFs group, Ox-LDL+PEMFs+HC067047 group, and Ox-LDL+ PEMFs+GSK1016790A group, n=5 independent experiments per group. b, LDH release level in the supernatant of HUVECs was detected by the LDH kit, n=5 independent experiments per group. c through l, Levels of IL-1β, IL-6, IL-18, MCP-1, and TNF-α in the supernatant of HUVECs or HAECs were detected by ELISA, n=5 independent experiments per group. All data represent biological replicates. The measured data were presented as the mean ± SEM. Statistical significance was assessed by one-way ANOVA with Tukey’s multiple comparison test. **p*<0.05，***p*<0.01，****p*<0.001，*****p*<0.0001.

**
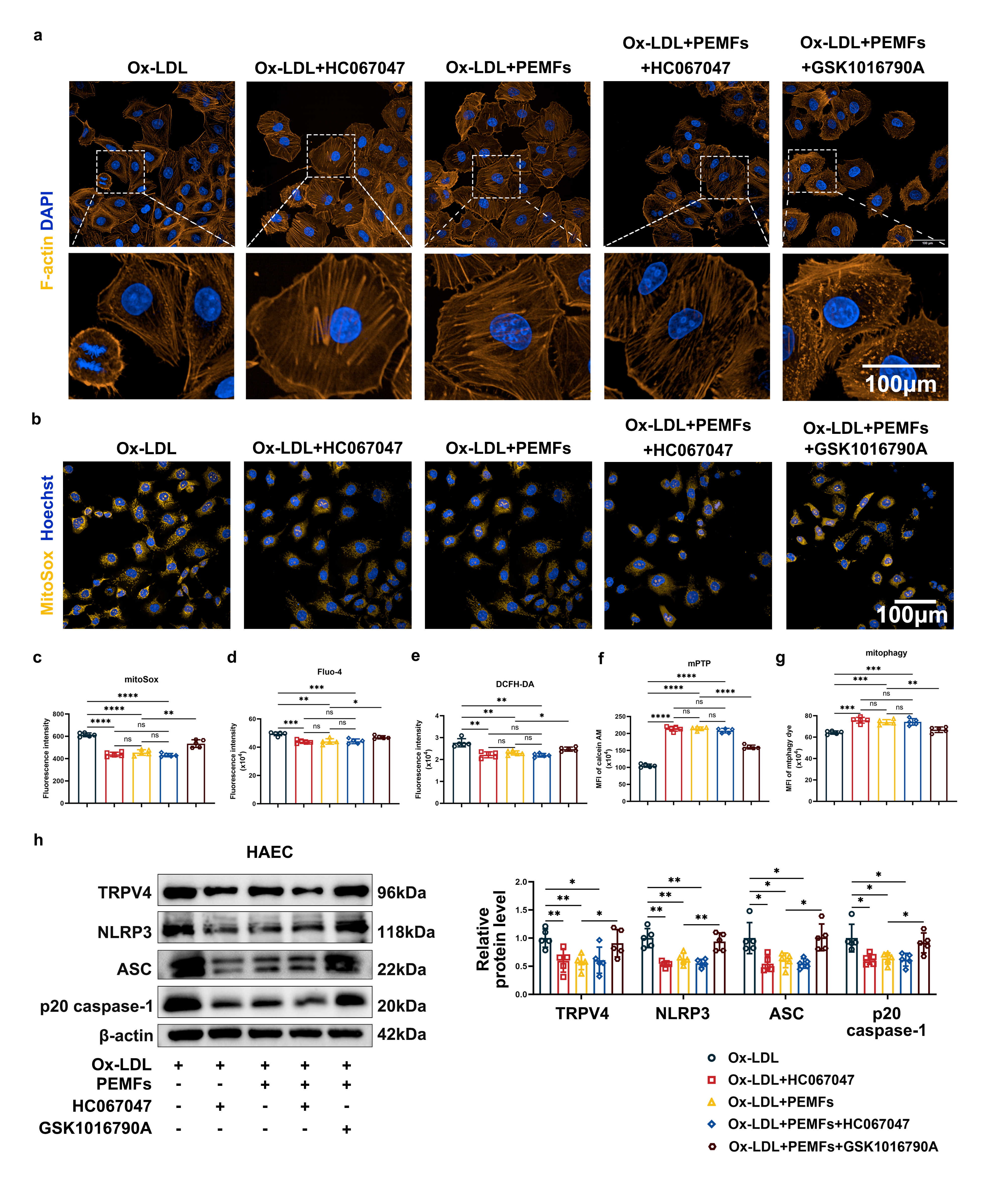
**

**Supplementary Figure 9. TRPV4 overactivation aggravated mitochondrial dysfunction and inflammation, contrasting with TRPV4 suppression which alleviated these pathological processes in HUVECs or HAECs.**

a, Cytoskeleton morphology was observed by F-actin (orange) and DAPI (blue) staining in HUVEC groups: Ox-LDL group, Ox-LDL+HC067047 group, Ox-LDL+PEMFs group, Ox-LDL+PEMFs+HC067047 group, and Ox-LDL+PEMFs+GSK1016790A group, n=5 independent experiments per group. Scale bar=100 μm. b and c, Mitochondrial ROS levels were detected by mitoSox, n=5 independent experiments per group. Scale bar=100 μm. d, Intracellular calcium ion levels were detected using Fluo-4 AM, n=5 independent experiments per group. e, Cellular superoxide anion levels were detected by DCFH-DA, n=5 independent experiments per group. f, mPTP opening was detected by the mPTP detection kit, n=5 independent experiments per group. g, Mitophagy was assessed by flow cytometry using a mitophagy detection kit, n=5 independent experiments per group. h. Western blot analysis of TRPV4, NLRP3, ASC, and p20 caspase-1 protein expression in HAECs, n=5 independent experiments per group. All data represent biological replicates. The measured data were presented as the mean ± SEM. Statistical significance was assessed by one-way ANOVA with Tukey’s multiple comparison test. **p*<0.05，***p*<0.01，****p*<0.001，*****p*<0.0001.


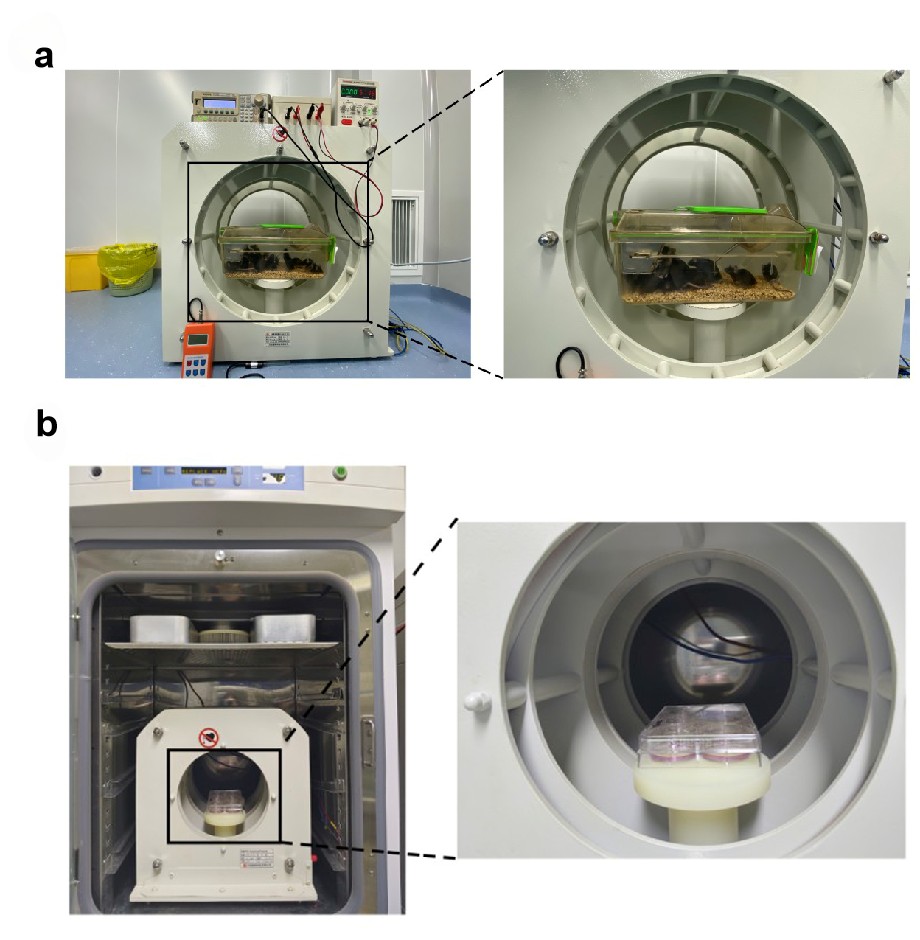


**Supplementary Figure 10. PEMFs generator.**

a. The PEMFs generator for *in vivo* experiments consists of a power supply, a pulse signal generator, a pulse signal amplifier, and two circular Helmholtz coils. These two circular Helmholtz coils exhibit identical thermal effects, enabling them to generate and provide an electromagnetic field with uniform distribution inside. Mouse cages were placed within the coils, with five mice housed in each cage. The generated hertz (frequency) and gauss (magnetic field intensity) levels were measured around the circumference of the cages. b. For the PEMFs generator used in *in vitro* experiments, the Helmholtz coils were installed inside a cell incubator. Thermal effects were excluded by monitoring the incubator temperature. Cell culture dishes or well plates were placed inside the coils, and the generated hertz and gauss levels were assessed around the perimeter of the culture vessels.

**Supplementary Table 1. Baseline differences between CHD and non-CHD patients.**

|  | **CHD (n=20)** | **non-CHD (n=20)** | ***p*** |
| --- | --- | --- | --- |
| Age (y) | 59.65 ± 8.92 | 59.30 ± 11.09 | 0.907 |
| Men (%) | 75.0% (15) | 70.0% (14) | 0.739 |
| TC (mmol/L) | 5.24 ± 1.38 | 3.73 ± 0.96 | <0.001 |
| TG (mmol/L) | 2.51 ± 1.32 | 1.20 ± 0.47 | <0.001 |
| HDL-C (mmol/L) | 1.06 ± 0.38 | 1.22 ± 0.31 | 0.142 |
| LDL-C (mmol/L) | 4.08 ± 1.89 | 2.35 ± 0.92 | <0.001 |
| GLU (mmol/L) | 7.70 ± 2.78 | 5.33 ± 0.91 | <0.001 |
| HbA1c (%) | 7.31 ± 2.07 | 5.23 ± 0.77 | <0.001 |
| hsCRP (mg/L) | 13.92 ± 16.39 | 1.73 ± 1.09 | <0.001 |
| LDH (IU/L) | 367.70 ± 194.93 | 216.70 ± 97.42 | 0.002 |
| AST (U/L) | 37.95 ± 34.52 | 22.95 ± 18.76 | 0.043 |
| ALT (U/L) | 34.05 ± 31.68 | 20.55 ± 15.21 | 0.069 |
| TBIL，μmol/L | 13.41 ± 7.25 | 14.69 ± 7.38 | 0.587 |
| DBIL，μmol/L | 4.13 ± 2.28 | 4.98 ± 2.75 | 0.279 |
| IBIL，μmol/L | 9.28 ± 5.61 | 9.71 ± 5.26 | 0.803 |
| CREA (μmol/L) | 88.36 ± 22.67 | 82.14 ± 18.63 | 0.342 |
| UREA (mmol/L) | 7.56 ± 2.20 | 5.97 ± 1.72 | 0.011 |

Abbreviations: TC, total cholesterol; TG, triglycerides; HDL-C, high-density lipoprotein cholesterol; LDL-C, low-density lipoprotein cholesterol; GLU, glucose; HbA1c%, glycated hemoglobin; hsCRP, high sensitivity C-reactive protein; LDH, lactate dehydrogenase; AST, aspartate aminotransferase; ALT, alanine aminotransferase; TBIL, total bilirubin; DBIL, direct bilirubin; IBIL, indirect bilirubin; CREA, creatinine.
